# Human amniotic mesenchymal stem cells-derived IGFBP-3, DKK-3, and DKK-1 attenuate liver fibrosis through inhibiting hepatic stellate cell activation by blocking Wnt/β-catenin signaling pathway in mice

**DOI:** 10.1186/s13287-022-02906-z

**Published:** 2022-06-03

**Authors:** Quan-Wen Liu, Yan-Min Ying, Jia-Xin Zhou, Wen-Jie Zhang, Zhao-xiao Liu, Bing-Bing Jia, Hao-Cheng Gu, Chu-Yu Zhao, Xiao-Hui Guan, Ke-Yu Deng, Hong-Bo Xin

**Affiliations:** 1 The National Engineering Research Center for Bioengineering Drugs and the Technologies, Institute of Translational Medicine, Nanchang University, No. 1299 Xuefu Road, Honggutan District, Nanchang, 330031 Jiangxi Province People’s Republic of China; 2 School of Life and Science, Nanchang University, Nanchang, 330031 People’s Republic of China; 3 Jiangxi Provincial Key Laboratory of Interdisciplinary Science, Nanchang University, Nanchang, 330031 People’s Republic of China; 4 Department of Obstetrics and Gynecology, The Second Affiliated Hospital of Nanchang University, Nanchang, 330006 People’s Republic of China; 5 Zhejiang Provincial Key Lab of Geriatrics, Department of Geriatrics, Zhejiang Hospital, Hangzhou, 310013 People’s Republic of China

**Keywords:** Liver fibrosis, Human amniotic mesenchymal stem cells, Hepatic stellate cell, Antibody array, Wnt/β-catenin signaling pathway

## Abstract

**Background:**

Liver fibrosis is an outcome of restoring process in chronic liver injury. Human amniotic mesenchymal stem cells (hAMSCs) derived from amniotic membrane have multilineage differentiation, immunosuppressive, and anti-inflammatory potential which makes them suitable for treating liver fibrosis. This study aimed to explore the effect and mechanism of hAMSCs on liver fibrosis.

**Methods:**

hAMSCs were transplanted into carbon tetrachloride (CCl_4_)-induced liver fibrosis mice via tail vein, and the effects of hAMSCs on hepatic fibrosis were assessed. The effects of hAMSCs and hAMSCs conditional medium (CM) on the activation of hepatic stellate cells (HSCs) were investigated in vivo and in vitro. Antibody array assay was used to identify the cytokines secreted by hAMSCs that may inhibit the activation of HSCs. Finally, the underlying mechanisms were explored by assessing IGF-1R/PI3K/AKT and GSK3β/β-catenin signaling pathways in the activated HSCs (LX-2) with hAMSCs and hAMSCs transfected with corresponding siRNAs.

**Results:**

Our results showed that hAMSCs possessed the characterizations of mesenchymal stem cells. hAMSCs significantly reduced liver fibrosis and improved liver function in mice by inhibiting HSCs activation in vivo. Both hAMSCs and hAMSC-CM remarkably inhibited the collagen deposition and activation of LX-2 cells in vitro. Antibody array assay showed that insulin-like growth factor binding protein-3 (IGFBP-3), Dickkopf-3 (DKK-3), and Dickkopf-1 (DKK-1) were highly expressed in the co-culture group and hAMSC-CM group compared with LX-2 group. Western blot assay demonstrated that IGFBP-3, DKK-3, and DKK-1 derived from hAMSCs inhibit LX-2 cell activation through blocking canonical Wnt signaling pathway.

**Conclusions:**

Our results demonstrated that IGFBP-3, Dkk3, and DKK-1 secreted by hAMSCs attenuated liver fibrosis in mice through inhibiting HSCs activation via depression of Wnt/β-catenin signaling pathway, suggesting that hAMSCs or hAMSC-CM provides an alternative therapeutic approach for the treatment of liver fibrosis.

**Supplementary Information:**

The online version contains supplementary material available at 10.1186/s13287-022-02906-z.

## Background

Liver fibrosis is a chronic disease resulting from repeated injuries of liver, caused by immune cytotoxicity, infection, metabolic disorders, and drug toxicity [1]. Liver fibrosis often leads to severe outcomes such as liver cirrhosis or even hepatocellular carcinoma which cause serious health problem worldwide [2]. Clinically, liver fibrosis is characterized by extracellular matrix (ECM) protein production and accumulation [3]. Hepatic stellate cells (HSCs), a kind of perisinusoidal non-proliferating cells, are the key player in the development of liver fibrosis. HSCs are silent in normal liver tissue, and they will be activated by different types of hepatic injury [4]. After activation, HSCs start to proliferate to myofibroblast-like cells which express alpha-smooth muscle actin (α-SMA), and produce ECM components and pro-inflammatory cytokines [5]. Currently, the most effective treatment for end-stage liver fibrosis is liver transplantation. However, lacking of transplant donors is a problematic for liver transplantation [6]. Thus, it is essential and urgent to seek a new treatment to control liver fibrosis by inhibiting the activation of HSCs.

Mesenchymal stem cells (MSCs) are a heterogeneous subset of stromal stem cells that can be isolated from many adult tissues, in which they are able to differentiate into the mesodermal lineage cells such as adipocytes, osteocytes, and chondrocytes, as well as other embryonic lineage cells [7]. In general, human MSCs express variable levels of CD29, CD105, CD90, CD73, and CD44, whereas they do not express the hematopoietic markers CD34, CD45, and the co-stimulatory molecules CD40, CD80, and CD86 [8]. MSCs have low immunogenic profile, anti-inflammatory function, high proliferation, high regenerative, and reparative potential, which make them extensively studied in clinical and preclinical trials [9]. Previous studies have demonstrated that MSCs-based therapy is a promising treatment strategy for liver fibrosis [10, 11]. A few studies support that MSCs can inhibit liver fibrosis by differentiating into hepatocyte-like cells in vivo [12]. In contrast, most of the studies hold the view that the therapeutic potential of MSCs in the treatment of liver fibrosis is predominantly based on their ability to secrete various trophic factors [13], such as interleukin-10 (IL-10) [14], milk fat globule-EGF factor 8 (MFGE8) [15], DKK-1 [16], fibroblast growth factor 4 (FGF4), hepatocyte growth factor (HGF) [17], and so on. The anti-fibrotic effects of these MSC-derived trophic factors can be distinguished by their direct or indirect effects on HSCs. The indirect anti-fibrotic effects are achieved by controlling immune cells, which subsequently inhibit the activity of HSCs, whereas the direct anti-fibrotic effects are mediated by inhibiting the activity of HSCs [13, 18]. In recent years, hAMSCs have been considered the stem cells with the most application prospect clinically. In comparison with other MSCs, hAMSCs have the great advantages that they can be obtained without invasive procedures and they have enormous proliferative capacity [19]. In our previous study, we have successfully isolated and identified hAMSCs [20, 21]. Many studies have been reported the potential effects of hAMSCs on liver fibrosis in animal models. However, the underlying mechanism is far from being elucidated.

In this study, we first labeled hAMSCs with green fluorescent protein (GFP) and characterized their morphology, multipotency, and growth potency. We observed that the transfection of GFP did not affect the characteristics of hAMSCs. In vivo, hAMSCs were evaluated for their effectiveness in liver fibrosis treatment using a CCl_4_-induced liver fibrosis mouse model. We found that hAMSCs transplantation via tail vein reduced collagen deposition, lessen fibrotic areas, and improved liver functions. In vitro, a transwell co-culture system and hAMSC-CM were used to assess the influences of hAMSC-secreted factors on the activation of HSCs. Antibody array assay was used to identify the differentially expressed cytokines which highly expressed in the coculture group and hAMSCs group compared with LX-2 group. The results showed that IGFBP3, DKK-3, and DKK-1 secreted from hAMSCs significantly inhibited HSCs activation through blocking Wnt/β-catenin signaling pathway.

## Materials and methods

### hAMSCs isolation and culture

Fresh amniotic membrane tissue was collected from the Department of Obstetrics and Gynecology, The First Affiliated Hospital of Nanchang University. hAMSCs were isolated and cultured in hAMSCs complete medium as previously described [20, 21].

Briefly, the amniotic membrane was firstly treated with 0.25% trypsin–EDTA (Thermo Fisher, Nanchang, China) to release human amniotic epithelial stem cells. Then, the amnion was washed three times with HBSS and digested with Collagenase IV (1 g/L, Thermo Fisher, Nanchang, China) on a rotator 40 min at 37 °C. Digestion was terminated by addition of medium containing 10% FBS. The single-cell suspension was filtered through a 70-μm cell strainer (BD Labware, Shanghai, China) and centrifuged at 1000 rpm for 5 min. The supernatant was discarded, and the cells were re-suspended with α-MEM medium (Thermo Fisher) containing 18% Chang B, 2% Chang C (Irvine Scientific), 10% FBS, 1% glutamine, and 1% penicillin/ streptomycin (Gibco). hAMSCs were then cultured in cell culture dishes (Corning, NY, USA) at a density of 5 × 10^4^ cells/cm^2^ at 37 °C with 5% CO_2_ atmosphere. This study was approved by the Ethics Committee of the First Affiliated Hospital of Nanchang University. The obtained tissue samples were only used for scientific research. Informed consent was obtained from the donators who voluntarily donated their placentas prior to their participation.

### Flow cytometry

Passage 3 hAMSCs were washed and resuspended at a concentration of 5 × 10^6^ cells/mL in staining buffer (PBS). Cells were incubated in the dark at 2–8 °C with FITC-conjugated antibodies against human CD29, CD90, CD45, HLA-DR, CD80, and CD40; phycoerythrin (PE)-conjugated antibodies against human CD73, CD105, CD34, HLA-ABC, and CD86; and their isotype controls (all from BD Biosciences). After 30 min, the cell suspensions were washed twice and resuspended in 200 μL PBS for flow cytometry (FACS Aria, BD Biosciences) using FLOWJO TM software (TreeStar, Inc., Ashland, OR, USA).

### Osteogenic and adipogenic differentiation

When hAMSCs (Passage 3) reached to 100% confluence in the six-well plate, OriCell™ human mesenchymal stem cell osteogenic differentiation medium (Cyagen Biosciences) was added to wells according to the manufacturer’s instruction. After 23 days of induction, Alizarin Red (pH 4.2, 40 mM) (Cyagen Biosciences) staining was performed to assess the differentiation potential for osteogenesis. For osteogenesis differentiation, hAMSCs were cultured with OriCell™ human mesenchymal stem cell adipogenic differentiation medium (Cyagen Biosciences, Shanghai, China) for 24 days to analyze the adipogenic differentiation. The differentiation potential of adipogenesis formation of intracellular lipid droplets was assessed by Oil red O (Cyagen Biosciences) staining.

### Lentiviral transduction

The lentiviral GFP expression vector pHBLV-IRES-ZsGreen-PGK-puro was purchased from Hanbio (Shanghai, China). hAMSCs were infected and selected as previously described [20]. Briefly, hAMSCs at passage 3 were infected with virus supernatant (multiplicity of infection 60) containing polyamine (6 mg/mL) for 24 h. Subsequently, puromycin (3 mg/mL; Sigma-Aldrich) was used to select GFP-positive cells. The percentage of GFP-labeled hAMSCs was determined by immunofluorescence every day. Once the percentage of GFP-labeled hAMSCs was higher than 95%, puromycin was removed from the culture medium.

### Liver fibrosis model induction and hAMSCs transplantation

Male C57BL/6 mice (8 weeks of age) were obtained from Changsha SLAC Laboratory Animal Company (Changsha, China, http://www.hnsja.com/). All animal experiments were performed according to institutional guidelines and approved by the Animal Care and Use Committee of Nanchang University. Liver fibrosis mice were induced by CCl_4_ olive oil solution (CCl_4_: olive oil = 1:9) via intraperitoneal injection at a concentration of 1 mL/kg twice per week for 6 consecutive weeks. Accordingly, the control group mice were subjected to the same concentration of olive oil (control group).

For the purpose of cell tracking and evaluation of the therapeutic efficacy of hAMSCs on liver fibrosis, 1.5 × 10^6^ GFP-labeled hAMSCs in 300 μL of PBS were injected into the tail vein of mice after 4 weeks of CCl_4_ treatment per week for 2 consecutive weeks (hAMSCs group). Mice injected with an equal amount of PBS without hAMSCs were used as the vehicle control for hAMSCs transplantation (PBS group).

### Whole body fluorescent imaging

After 6 weeks of CCl_4_ treatment, mice were euthanized and their liver, heart, spleen, lung, kidney, pancreas, and brain were harvested and visualized with whole-body fluorescent imaging system (LB983; Berthold, Germany).

### Histopathology and sirius red staining

Liver tissues were processed for paraffin embedding by slicing into 5-μm sections. After deparaffinization and rehydration, the sections were rinsed in PBS and then incubated in a 3% H_2_O_2_ solution to block the endogenous peroxidase. After incubation with 5% BSA for 30 min to block non-specific antibody-binding sites, the samples were stained with primary antibody against α-SMA (1:200, mouse monoclonal, Abcam), TGF-β (1:200, rabbit polyclonal, Abcam), Anti-human Nuclei Antibody MAB1281 (1:200, mouse monoclonal, Merck), and CD90 (1:250, rabbit monoclonal, Abcam) at 4 °C overnight. The samples were rinsed with PBS twice and incubated with a HRP-conjugated goat anti-mouse/rabbit secondary antibody (MaiXin biotechnologies, China) followed by visualization with 3,3-diaminobenzidine tetrahydrochloride (MaiXin biotechnologies). Finally, the sections were stained with hematoxylin and examined under a light microscope (IX83; Olympus, Japan).

For Sirius Red Staining, the sections were stained with Picro-Sirius red solution (Solarbio, Beijing, China), for 30 min, washed with 100% ethanol, dried in an oven at 60 °C, cleared in xylene for 5 min, mounted in a neutral balsam mounting medium (Solarbio), and examined under a light microscope (Olympus).

### Biochemical analysis

After 6 weeks of CCl_4_ treatment, blood samples were collected from each mouse and then centrifuged at 3500 rpm for 30 min. The serum was collected for measurements of total bilirubin (TBIL), alanine aminotransferase (ALT), aspartate aminotransferase (AST), albumin (ALB), and alkaline phosphatase (ALP) with an automated biochemical analyzer (Abbott Aeroset, Abbott Laboratories, Chicago, IL, USA). All samples were run in triplicate.

### Protein extraction and Western blot analysis

The liver tissues and LX-2 cells in each group were lysed with radioimmunoprecipitation assay (RIPA) lysis buffer (Solarbio, Beijing, China) supplemented with complete protease inhibitor cocktail (Roche, Mannheim, Germany) to extract the total proteins. The suspension was centrifuged at 13,000 rpm at 4 °C for 10 min to remove insoluble debris and chromosomal DNA, and the supernatant containing proteins was collected. The total protein concentration was quantified using the BCA Protein Assay Kit (Solarbio, Beijing, China) and then mixed with a loading buffer (Solarbio, Beijing, China) and boiled at 100 °C for 10 min. In total, 30 μg of total protein was run on 10% denaturing SDS-PAGE gels and then transferred to nitrocellulose membranes (BioRad), which were incubated with primary antibodies anti-GAPDH (G-9) (1:1000, mouse monoclonal, Santa Cruz), anti-α-SMA (1A4) (1:2000, mouse monoclonal, Abcam), anti-TGF-β (full length) (1:1000, rabbit polyclonal, Abcam), anti-Collagen I (full length) (1:1000, mouse monoclonal, Abcam), anti-Collagen III (EPR17673) (1:1000, rabbit polyclonal, Abcam), anti-PCNA (PC10) (1:1000, mouse monoclonal antibody, Abcam), anti-Bcl-2 (100/D5) (1:1000, mouse monoclonal, Abcam), β-catenin (E247) (1:1000, rabbit polyclonal, Abcam), PPAR-γ (EPR18516) (1:2000, rabbit monoclonal, Abcam), GSK3β (Y174) (1:1000, mouse monoclonal, Abcam), P-GSK3β (S9) (1:1000, rabbit polyclonal, Abcam), β-catenin (E247) (1:5000, rabbit monoclonal, Abcam), DKK-3 (EPR15611) (1:1000, rabbit monoclonal, Abcam), DKK-1 (B-7) (1:1000, mouse monoclonal, Santa Cruz), IGFBP-3 (EPR18680-153) (1:1000, rabbit monoclonal, Abcam), AKT (1F7E10) (1:1000, mouse monoclonal, Abcam), P-AKT (18F3.H11) (1:1000, mouse monoclonal, Abcam), PI3K (C73F8) (1:1000, rabbit polyclonal, CST), P-PI3K (Tyr458/Tyr199) (1:1000, rabbit polyclonal, CST), IGF-1R (D23H3) (1:1000, rabbit monoclonal, CST), P-IGF-1R (19H7) (1:1000, rabbit monoclonal, CST), ERK1/2 (EPR17526) (1:5000, rabbit monoclonal, Abcam), P-ERK1/2 (EPR19401) (1:1000, rabbit monoclonal, Abcam), JNK (EPR18841-95) (1:1000, rabbit monoclonal, Abcam), and P-JNK (EPR5693) (1:1000, rabbit monoclonal, Abcam) at 4 °C overnight. Blots were detected with horseradish peroxidase (HRP)-conjugated goat anti-rabbit or rabbit anti-mouse secondary antibody (Invitrogen) for 1 h at room temperature. Images were quantified using the Super Signal West Pico or Femto chemiluminescent detection system (Pierce).

### In vitro co-culture experiment

LX-2 cells were purchased from ATCC and cultured in six-well plates with RPMI 1640 supplemented with 10% FBS and 1% penicillin/streptomycin (LX-2 complete medium, all from Thermo Fisher) at a density of 1.5 × 10^5^ cells/well. For control group, LX-2 cells were cultured with 3 mL LX-2 complete medium. For co-culture group, a co-culture transwell chamber (24 mm diameter, 0.4 μm poresize; Corning) was used to assess the effects of hAMSCs on activated LX-2 cells. LX-2 cells were seed in the lower chamber with 2 mL LX-2 complete medium at a density of 1.5 × 10^5^ cells/well, and the hAMSCs were seeded in the upper compartment at a 1:1 ratio with LX-2 in 1 mL of LX-2 complete medium. For hAMSC-CM group, LX-2 cultured in the LX-2 complete medium supplemented with 10% hAMSC-CM (10 ×).

### Annexin V-PI apoptosis assay

For the apoptosis assays, cells were collected from each sample and resuspended in 100 μL Annexin V binding solution containing 5 μL Annexin V-FITC and 5μL propidium iodide (PI) solution (Dojindo). After incubation for 15 min at room temperature, cells were washed with PBS, centrifuged at 1000 rpm for 5 min, and resuspended in 400 μL Annexin V Binding Buffer. The apoptosis assays were run and analyzed with BD Jazz.

### CCK8 assay

Cell proliferation was evaluated at indicated time points using the CCK-8 kit (Dojindo Laboratories, Kumamoto, Japan), following the manufacturer’s protocol. CCK-8 reagent (10%) was added to each well for 3 h at 37 °C. Viability was evaluated by measuring the absorbance at a 450-nm wavelength with using a microplate spectrophotometer (BioRad).

### Cytokine antibody array

The experiment was divided into three groups. The control group and co-culture group were prepared as previously described (in vitro co-culture experiment). For hAMSCs group, hAMSCs were cultured with 3 mL complete medium. Human cytokine antibody array (GSH-CAA-440, RayBiotech, Norcross, GA, USA) was used to measure the expressions of 440 cytokines in culture supernatants of different groups at 48 h after culturing, according to the manufacturer’s instructions.

### Small interfering RNA (siRNA)-mediated silencing of IGFBP3, DKK-3, and DKK-1 expression

The siRNA targeting IGFBP3, DKK-3, and DKK-1 were purchased from Ribo-Bio (Guangzhou, China). hAMSCs were transfected with siIGFBP3, siDKK-3, or siDKK-1 at a final concentration of 50 nM using riboFECT™ CP Reagent (RayBiotech, Guangzhou, China) according to the manufacturer's instructions. hAMSCs transfected with only transfection reagent (without siRNA) were used as controls.

### Statistical analysis

The results are presented as average value ± standard deviation (SD). Student’s t test was used for analysis between two groups. One-way analysis of variance (ANOVA) was used to compare data among three or more groups. Differences with a *P*-value of < 0.05 were considered statistically significant.

## Results

### Characterization of hAMSCs

**Fig. 1:**
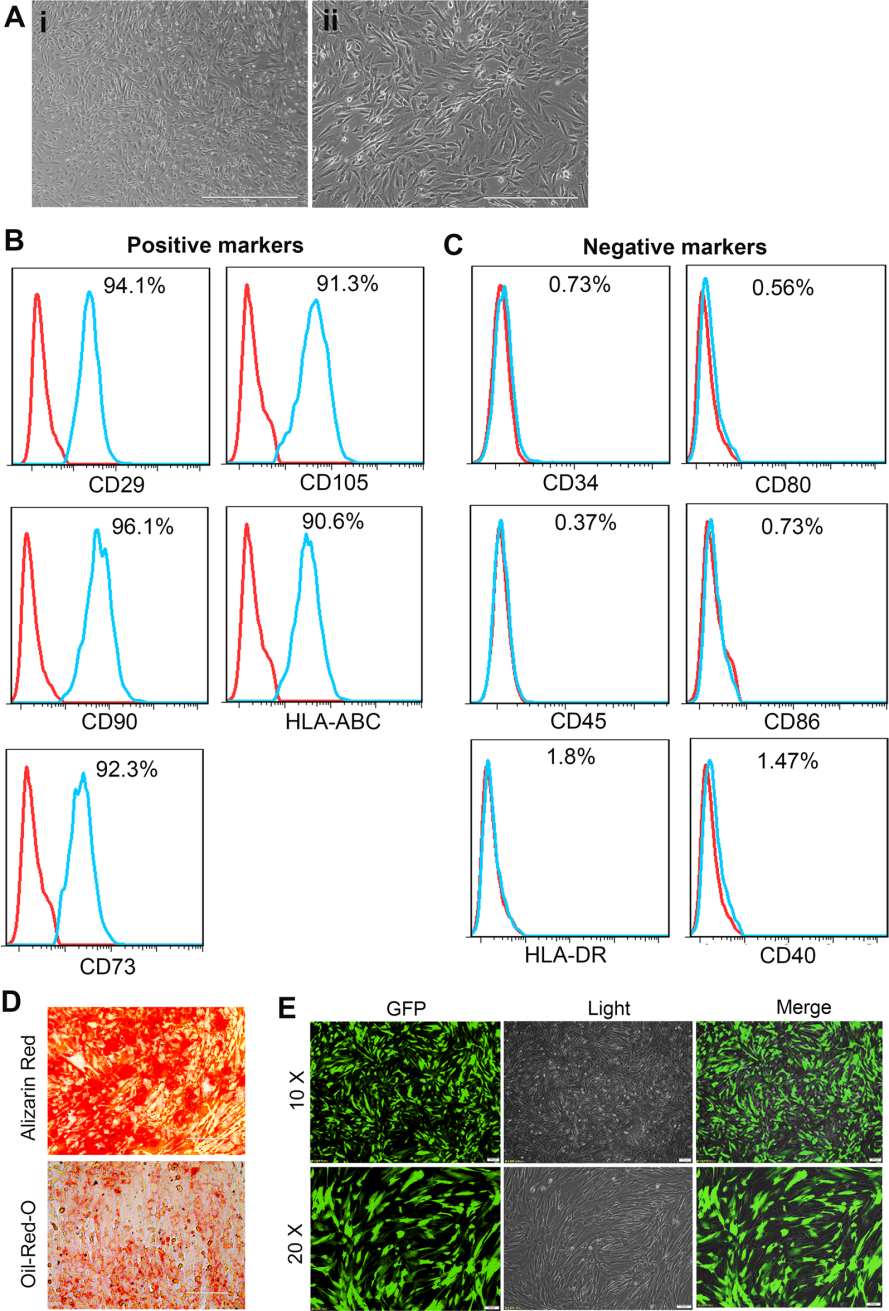
Morphological and immunophenotypic analysis of hAMSCs. **A** Morphological appearance of cultured hAMSCs. (1) Scale bar = 1000 μm, (2) scale bar = 400 μm. **B** Flow cytometry analysis indicated the positive expression of CD29, CD90, CD73, CD105, and HLA-ABC in hAMSCs. **C** Flow cytometry analysis indicated the negative expression of CD34, CD45, HLA-DR, CD80, CD86, and CD40 on cultured hAMSCs. **D** Osteogenic and adipogenic differentiation of hAMSCs was demonstrated by staining with Alizarin Red and oil red O staining, respectively **E** Representative images of cultured GFP-labeled hAMSCs

hAMSCs showed the plastic adherence properties and spindle-shaped morphology which are the primary characteristics of MSCs (Fig. 1A). Flow cytometric analysis revealed that hAMSCs expressed CD29, CD90, CD73, CD105, and HLA-ABC markers and did not express hematopoietic markers CD34 and CD45 (Fig. 1B, C). hAMSCs also lacked the expression of HLA-DR and HLA-ABC co-stimulates molecules CD80, CD86, and CD40, indicating that hAMSCs have low immunogenicity (Fig. 1C). In vitro, hAMSCs are able to differentiate into osteoblasts and adipocytes under osteogenic and adipogenic differentiation conditions (Fig. 1D). We previously reported that hAMSCs had no tumorigenicity in vitro and in vivo [21]. For cell tracking, the hAMSCs were labeled with GFP by lentiviral infection. As shown in Fig. 1E, more than 90% of hAMSCs were GFP-positive after puromycin selection. In addition, our previous results showed that the transfection of GFP did not affect the characteristics of hAMSCs [20].

### hAMSCs injected with tail vein reduced liver fibrosis and improved liver functions in mice with liver fibrosis induced by CCl_4_

**Fig. 2:**
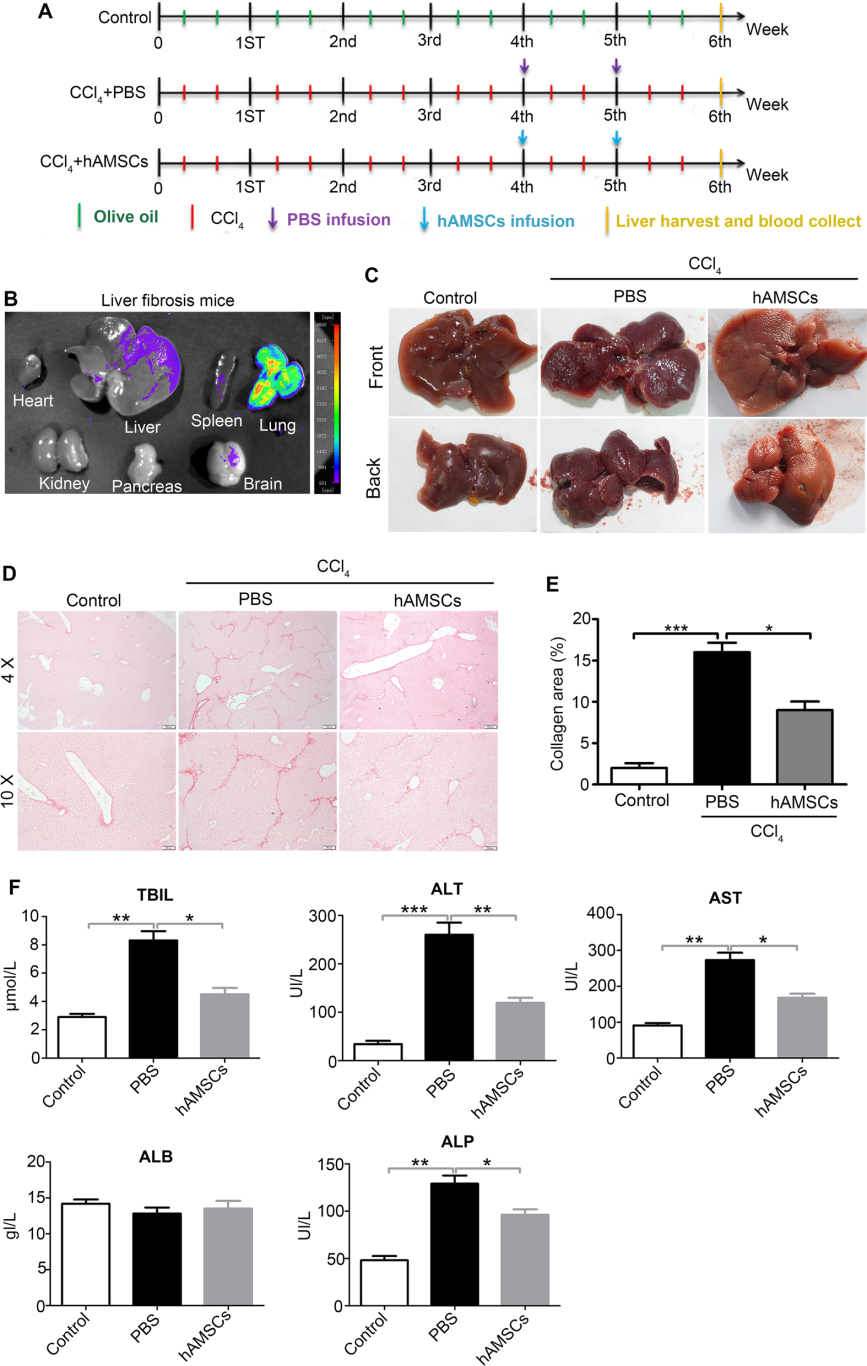
hAMSCs alleviates liver fibrosis and improves liver function in liver fibrosis mice. **A** The schematic diagram represents the experimental design regarding the induction of fibrosis and cell administration. **B** The distribution of GFP-labeled hAMSCs in vivo of liver fibrosis mice on day 7 or day 14 after transplantation. hAMSCs mainly distribution in the lung and liver. **C** The representative images of gross morphology of livers of control, PBS, and hAMSCs group. **D** Sirius Red Staining showing the degree of fibrosis in mice from different groups. **E** Quantitative analysis of the collagen area percentage in livers of different groups as shown in (**D**). **F** The serum levels of TBIL, ALT, AST, ALB, and ALP in each group. *P < 0.05, *P < 0.01, ***P < 0.001

To explore the effect of hAMSCs on liver fibrosis, a mouse model with liver fibrosis was induced by CCl_4_. The GFP-hAMSCs (1.5 × 10^6^ cells in 300 μL PBS) or PBS (300 μL) was intravenously injected in the 4th and 5th week after CCl_4_ treatment (Fig. 2A). The heart, liver, spleen, lung, kidney, pancreas, and brain were harvested at the 6th week after CCl_4_ treatment and visualized with whole-body fluorescent imaging system. As shown in Fig. 2B, partial hAMSCs migrated into injured liver. The liver of mice in control group had a smooth, soft texture, and uniform surface. In contrast, the liver of PBS group had more fibrous nodules and was less ruddy on the surface. Compared to the PBS group, the livers of mice in the hAMSCs group were smoother and softer than those of mice in the PBS group (Fig. 2C), indicating that the hepatic pathological changes were significantly ameliorated in the hAMSCs group. Sirius Red Staining showed that the liver tissue sections from PBS group exhibited focal fibrosis, confirming the successful establishment of liver fibrosis in mice. Moreover, Sirius Red Staining also showed that hAMSCs markedly reduced collagen deposition and lessen fibrotic areas compared with control group (Fig. 2D, E). In addition, hAMSCs significantly decreased serum levels of TBIL, ALT, AST, and ALP, and elevated the serum level of ALB in mice treated with hAMSCs compared with PBS group (Fig. 2F, Table 1). These results indicate that hAMSCs effectively inhibited liver fibrosis and improved liver function in mice with liver fibrosis.

**Table 1:** hAMSCs transplantation improved the liver function of CCl_4_-induced liver fibrosis mice

	TBIL (μmol/L)	ALT (U/L)	AST (U/L)	ALB (g/L)	ALP (U/L)
Control	2.9 ± 0.4	34 ± 12	91 ± 11	14.2 ± 1	48 ± 8
CCl_4_(6W) + PBS(2W)	8.1 ± 1.1	260 ± 43	273 ± 36	12.8 ± 1.5	129 ± 15
CCl_4_(6W) + hAMSCs (2W)	4.5 ± 0.8	119 ± 19	168 ± 20	13.5 ± 1.9	96 ± 10

**Fig. 3:**
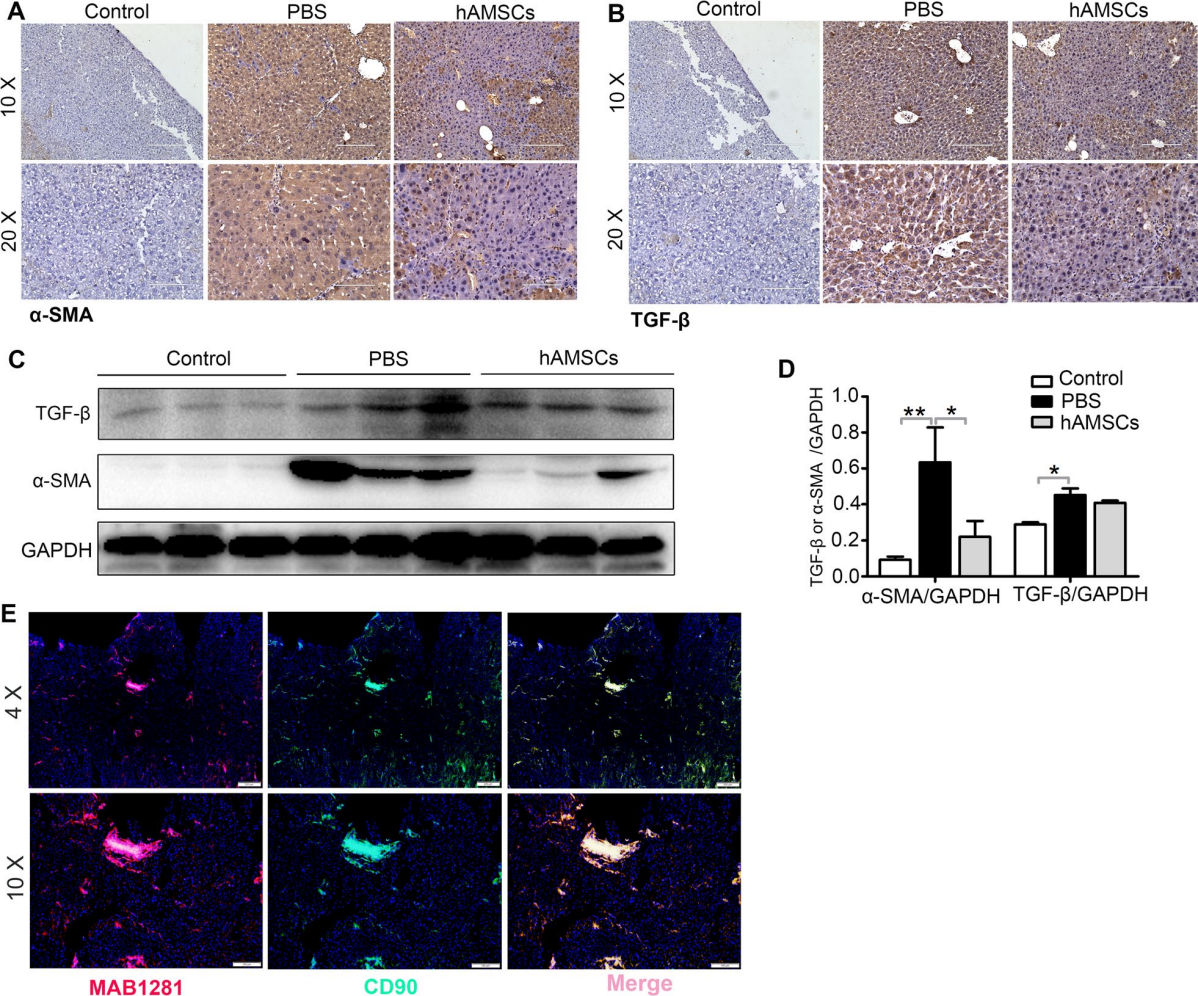
hAMSCs inhibit HSCs activation in liver fibrosis mice through paracrine mechanisms. **A**, **B** Immunohistochemical staining of α-SMA and TGF-β expressing cells in liver tissues of control, PBS, and hAMSCs group. **C** The protein expression levels of α-SMA and TGF-β in liver tissues of different groups were analyzed by western blot. **D** Quantitative analysis of the expression of α-SMA and TGF-β in liver tissues as in C. **E** The liver tissues sections of different groups were co-stained with MAB1281 and CD90 and imaged by confocal microscope. *P < 0.05, *P < 0.01, ***P < 0.001

Positive expression of α-SMA serves as a marker for HSC activation [1], and TGF-β is an important factor for induction of liver fibrosis [22]. Immunohistochemical staining and western blot results showed that the expressions of α-SMA and TGF-β in hAMSCs groups were lower than that in PBS group (Fig. 3A–D). Antibody to human-specific nuclei (MAB1281) was used to verify whether hAMSCs were differentiated into other kinds of cells in mouse liver tissues. The immunostaining analysis showed that the MAB1281-positive cells also expressed CD90, suggesting that the positive cells in mouse liver tissues represented the hAMSCs. Taken together, these results suggested that the protection of hAMSCs on liver fibrosis may be related to suppression of HSCs activation.

### hAMSCs inhibited HSCs activation by their paracrine mechanism in vitro

**Fig. 4:**
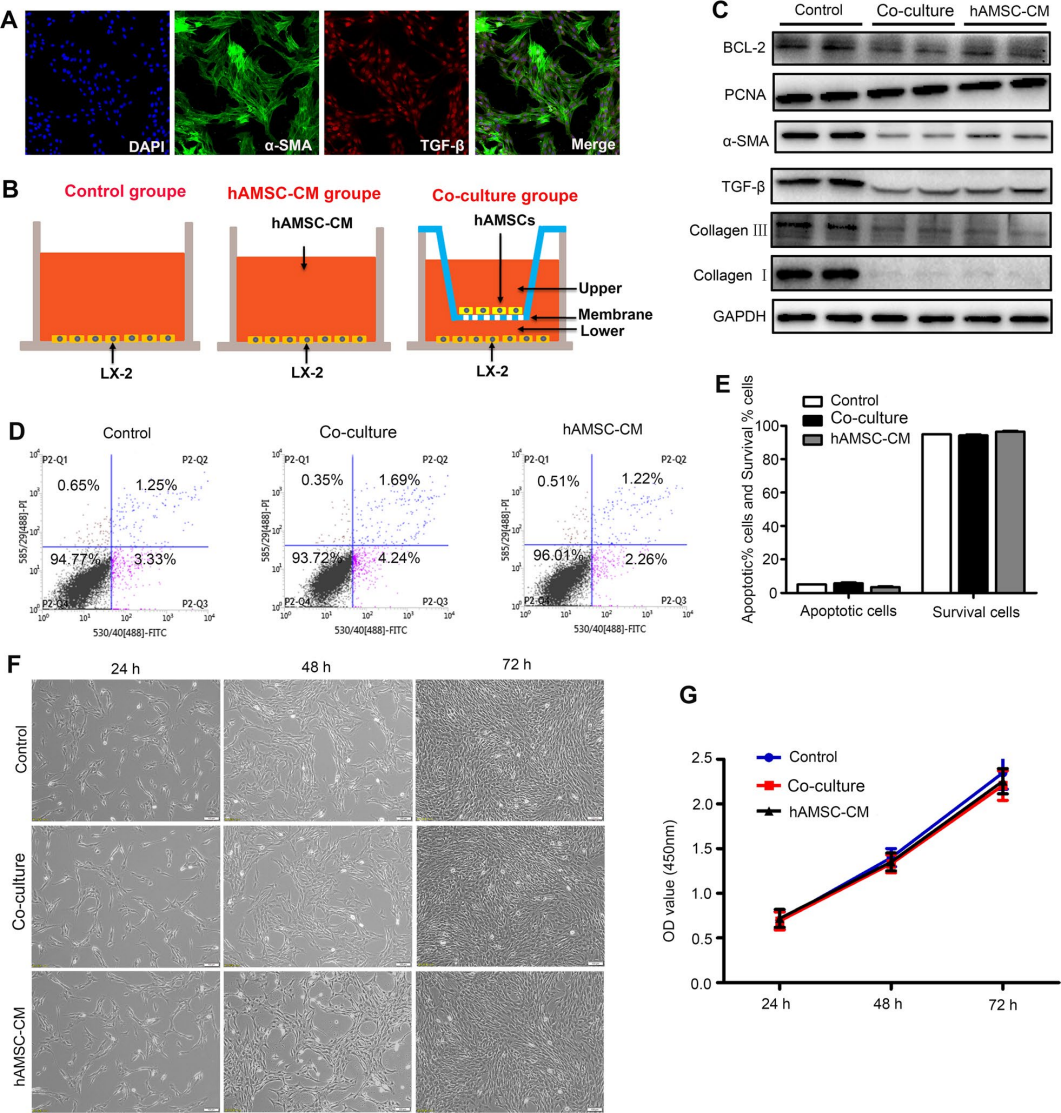
Effects of hAMSCs and hAMSC-CM on activation, apoptosis, and proliferation in LX-2 cells. **A** The expression levels of α-SMA and TGF-β in LX-2 cells were determined by immunofluorescence staining. **B** Schematic diagram of the control group, hAMSC-CM group, and co-culture group. **C** LX-2 cells were treated with normal medium (control), hAMSCs, and hAMSC-CM. The expression levels of Collagen I, Collagen III, TGF-β, α-SMA, PCNA, and Bcl-2 in LX-2 cells of different groups were analyzed by western blot after 48 h of treatment. **D** The apoptosis of cells was assessed by FACS. **E** Quantitative analysis of the percentage of apoptotic cells and survival cells as shown in (**D**). **F** Representative images of LX-2 cells observed under a light microscope after cultured with normal medium, hAMSCs, or hAMSC-CM for 24, 48, and 72 h. **G** CCK-8 assay for cell proliferation of LX-2 cells in different groups at different time points

LX-2 is an immortalized stellate cell line which is commonly used to mimic activated stellate cells in vitro [23]. As shown in Fig. 4A, the LX-2 cells highly expressed α-SMA and TGF-β, indicating that LX-2 cells were in an activated state. To further investigate the effects of hAMSCs on the apoptosis, proliferation, and activation of HSCs, LX-2 cells were co-cultured in 1:1 ratio with hAMSCs or treated with 10% hAMSC-CM (Fig. 4B). In addition, western blot results showed that after 48 h of treatment, hAMSCs or hAMSC-CM significantly reduced the expression levels of collagen I, collagen III, TGF-β, and α-SMA compared with the control group (Fig. 4C), suggesting that hAMSCs or hAMSC-CM will be able to inhibit the collagen deposition and activation of LX-2 cells. By contrast, there were no significant differences for the expressions of proliferation-related protein PCNA and apoptotic-related protein Bcl-2 between control, coculture, and hAMSC-CM group (Fig. 4C). Furthermore, Annexin V-PI apoptosis assay and CCK-8 assay results showed that there was no significant difference in the apoptosis (Fig. 4D, E) and proliferation (Fig. 4F, G) of LX-2 cells co-cultured with hAMSCs or hAMSC-CM compared with that of the control group, further confirming that hAMSCs or hAMSC-CM had no effects on the apoptosis and proliferation of LX-2 cells in vitro. Taken together, these data indicated that hAMSCs inhibited LX-2 cell activation in vitro by their paracrine mechanism since both hAMSCs or hAMSC-CM exerted an equal effect on the inhibition.

### There were certain cytokines with differential expression between LX-2, hAMSCs, and LX-2/hAMSCs co-culture group

**Fig. 5:**
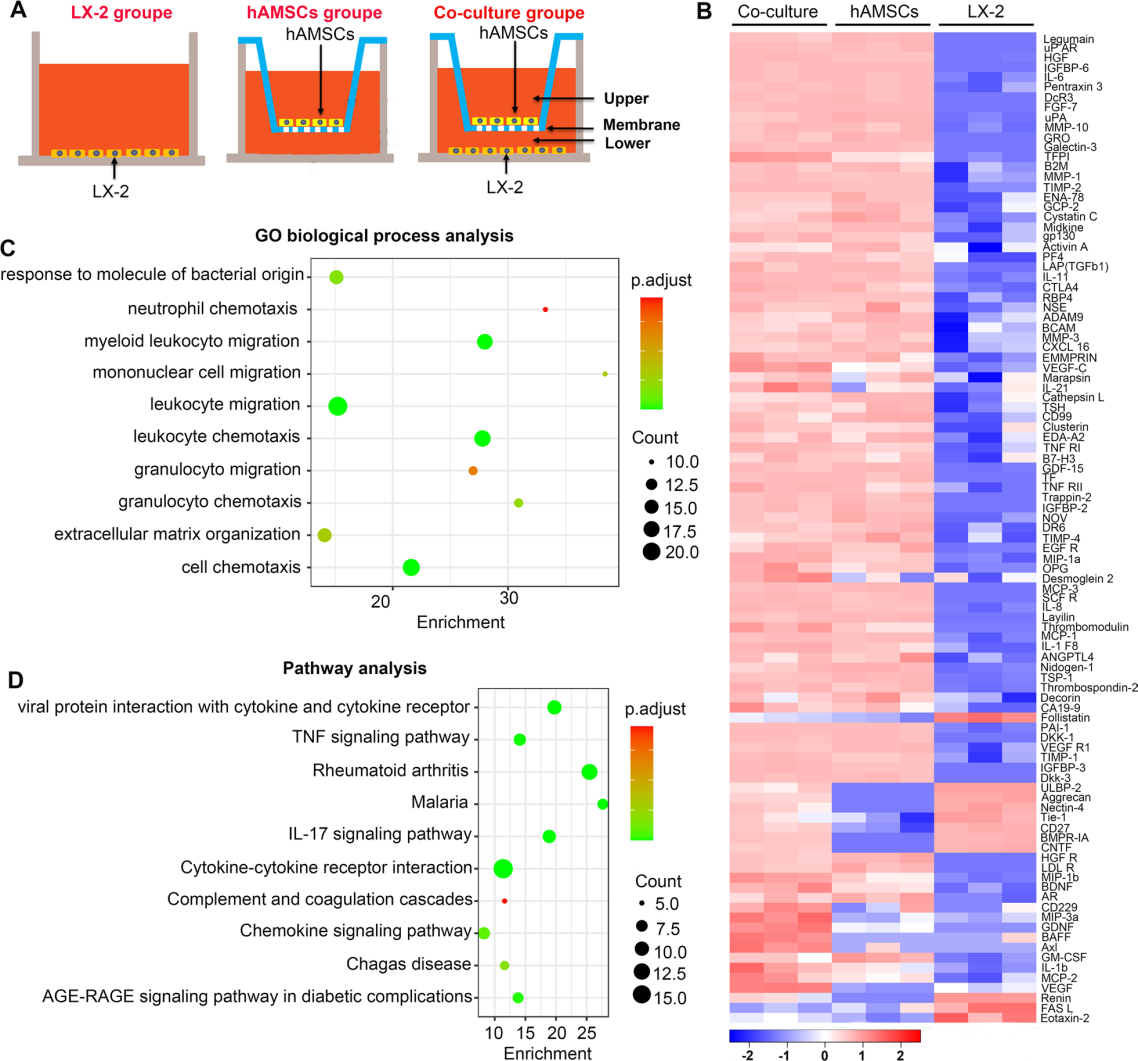
Cytokine expression profile of LX-2, hAMSCs, and LX-2/hAMSCs co-cultures. **A** Schematic diagram of the LX-2 group, hAMSCs group, and LX-2/hAMSCs co-culture group. **B** Hierarchical clustering of differentially expressed cytokines is shown in a heatmap. **C** The top 10 GO biological process terms of differentially expressed cytokines. **D** Top 10 functional pathways associated with these differentially expressed cytokines through KEGG analysis

To identify molecules involved in mediating the suppressive effects of hAMSCs on LX-2 cell activation, an antibody array was used to examine cytokine levels in the culture supernatants of the LX-2, hAMSCs, and co-culture group (Fig. 5A). Among 440 cytokines evaluated, the expressions of some cytokines were undetectable or extremely low, and several cytokines were differently expressed in the hAMSCs group, co-culture group, and LX-2 group (Additional file 1: Table S1). The differential expressions of cytokines were visualized in a heat map (Fig. 5B). To identify the biological characteristics of the cytokines with differential expression, the GO and pathway enrichment analyses were performed. Top 10 enriched terms in biological process categories and top 10 enriched pathways are shown in Fig. 5C, D. Because both hAMSCs and hAMSC-CM could inhibit the activation of LX-2 cells, we speculated that the molecules involved in the suppressive effect should highly express in the co-culture group and hAMSCs group but low express in the LX-2 group. As shown in Table 2, there were 56 cytokines that were highly expressed in the co-culture group and hAMSCs group compared with LX-2 group. Among them, IGFBP-3, PAI-1, TSP-1, VEGF R1, DKK-3, TFPI, ANGPTL4, Thrombospondin-2, TIMP-1, Nidogen-1, and DKK-1 showed the highest expression levels in the co-culture group and hAMSCs group.

**Table 2:** The differentially expressed cytokines which high expressed in the co-culture group and hAMSCs group but low expressed in the LX-2 group

Cytokines	Concentration (pg/mL)		
	Mean_Coculture	Mean_hAMSCs	Mean_LX-2
IGFBP-3	56,932.3678	45,401.20049	0
PAI-1	51,027.64547	47,508.89115	30.67226692
TSP-1	40,421.80083	46,888.60088	2083.020038
VEGF R1	33,031.62889	44,504.7282	38.3324137
Dkk-3	33,000.73426	37,200.47249	0
TFPI	24,775.95059	1248.839624	0.619501692
ANGPTL4	23,829.98389	28,309.84487	4435.142538
Thrombospondin-2	20,881.28501	19,240.45644	1444.047002
TIMP-1	19,493.73353	11,697.53576	35.7873645
Nidogen-1	17,032.03501	19,445.91645	3820.074253
DKK-1	12,279.79182	11,930.42348	30.32114266
TIMP-2	11,249.62613	9685.848476	4.123643825
B2M	7678.843668	8007.653905	54.90541142
IGFBP-6	6284.869031	7002.614248	0
Decorin	5473.517788	6627.550399	2902.522066
PF4	5442.824963	4101.662534	133.646572
MMP-1	4936.144319	4346.030104	29.940655
IL-6	3975.143781	3921.847936	3.180008485
uPAR	3409.932352	3931.905869	0
CA19-9	3381.193656	2881.310063	1102.400163
Legumain	3235.955439	5166.152115	0
Pentraxin 3	3201.645922	3614.372483	6.934564818
HGF	3076.933415	2381.403175	0.079359219
ENA-78	2450.070326	4644.609358	46.0612987
LAP(TGFb1)	2263.679658	2133.156608	48.40492963
RBP4	2062.022266	1864.392887	34.75823158
gp130	1782.554108	1057.827792	26.06557417
CTLA4	1552.47379	1143.796665	21.52788958
IL-11	1469.715536	1297.520964	37.76951402
GRO	1395.887705	2214.43841	0
Galectin-3	1380.709054	1383.173842	0
IL-21	1209.640755	542.5699298	353.4675817
MMP-3	1107.167868	978.3551104	41.95096733
MMP-10	1027.558414	930.4165444	0.748626507
NSE	1019.089452	1201.459356	53.20761037
DcR3	954.7013785	1040.192828	0
GCP-2	949.5248561	1984.953832	57.43712661
IL-1 F8	862.9746155	399.3486518	2.446001288
FGF-7	778.3293691	865.6898357	0
uPA	746.3596929	1128.005703	0.352235285
BCAM	667.6812718	851.7541275	95.80577077
MCP-1	595.2255118	595.3578171	2.784970381
ADAM9	588.5333371	918.4646244	104.9219854
Thrombomodulin	560.3082502	144.2274444	0
Activin A	560.1542887	1357.888614	138.9795245
Midkine	540.038388	774.612734	11.18265853
MCP-3	536.2496514	577.5162488	0
Cystatin C	489.9678272	855.6066023	12.78700818
SCF R	483.8017963	402.8085761	0
EMMPRIN	412.651994	347.3121665	76.69804627
Marapsin	397.2022369	357.5719418	106.3995523
CXCL16	384.2995872	611.1009215	23.84031576
TNF RI	330.4061239	191.8122683	5.801326368
IL-8	297.1578354	257.0999096	0.71535184
VEGF-C	217.9682498	121.5056078	45.60550059
Layilin	215.6953522	265.9848876	0

### hAMSCs-derived Dkk-3 and Dkk-1 inhibited HSCs activation through blocking Wnt/β-catenin signaling pathway

**Fig. 6:**
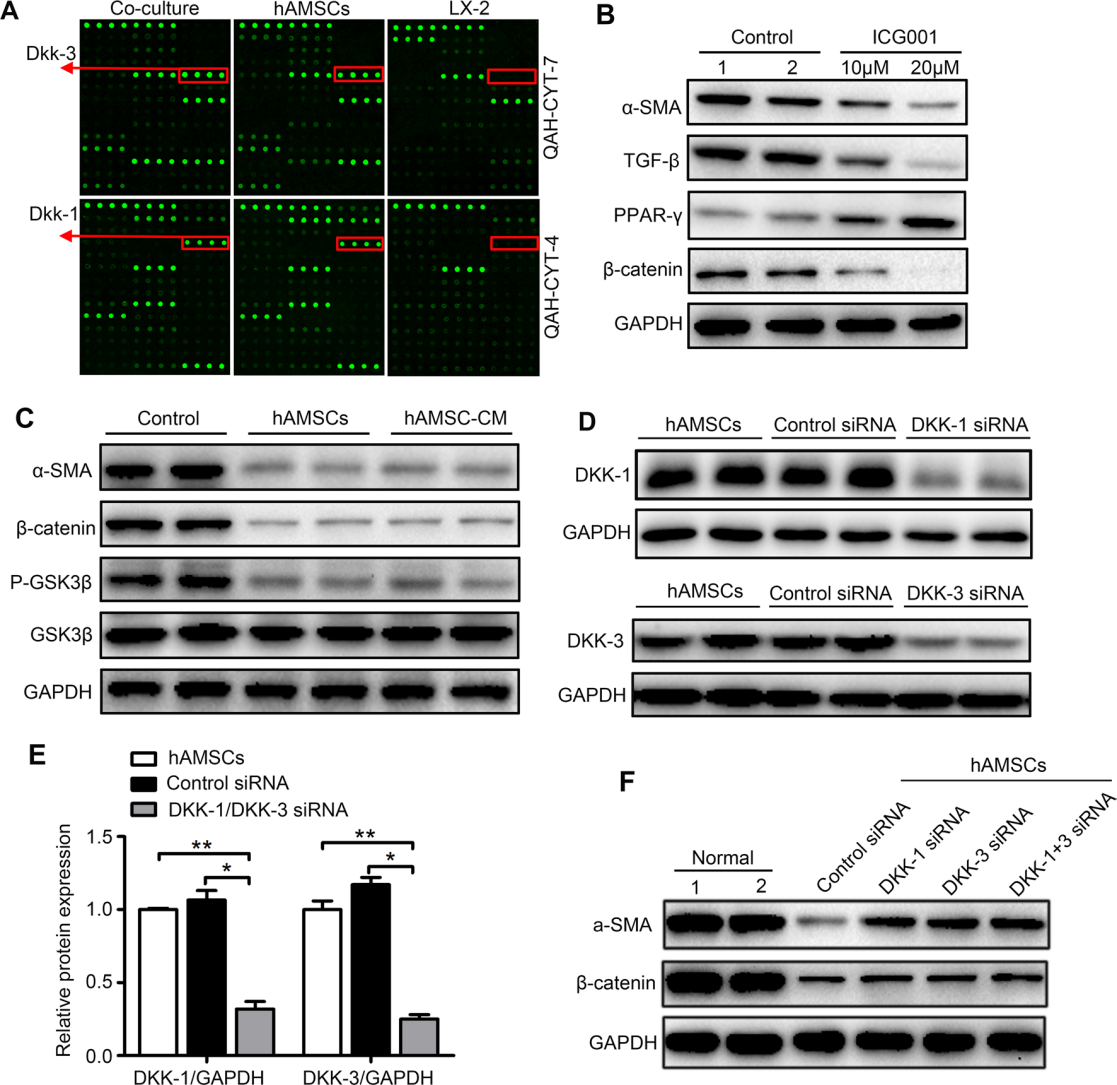
DKK-3 and DKK-1 derived from hAMSCs inhibit LX-2 cell activation through blocking canonical Wnt signaling pathway. **A** Representative array images are shown (*n* = 4). DKK-3 and DKK-1 are highlighted with red boxes and arrows, accordingly. **B** Western blot detection of β-catenin, PPAR-γ, TGF-β, and α-SMA protein expression in LX-2 after ICG001 treatment. The normal LX-2 was used as control. **C** LX-2 cells were treated with normal medium (control), hAMSCs, and hAMSC-CM. The expression levels of GSK3β, P-GSK3β, β-catenin, and α-SMA in LX-2 cells of different groups were analyzed by western blot after 48 h of treatment. **D** LX-2 was cultured for 48 h in the presence of control siRNA or DKK-1/DKK-3 siRNA. Cell lysates were analyzed by western blot using antibodies against DKK-1 and DKK-3. **E** Quantitative analysis of the expression of DKK-1 and DKK-3 in hAMSCs cells of different groups as in (**D**). **F** Western blot analysis showed that the accumulation of β-catenin and the expression of α-SMA were increased in DKK-1 siRNA, DKK-3 siRNA, and DKK-1 + DKK-3 siRNA group when compared with control siRNA group

Dkk-3 and Dkk-1, which have been proved to be highly expressed in the co-culture group and hAMSCs group compared to LX-2 group (Table 2 and Fig. 6A), are two antagonists of Wnt/β-catenin signaling. In order to confirm whether the Wnt/β-catenin pathway is necessary for the activation of HSCs, the activated LX-2 cells were treated with or without β-catenin inhibitor ICG001 (10 μM and 20 μM). The results showed that ICG001 inhibited the expression of β-catenin, TGF-β, and α-SMA and increased the expression of PPAR-γ, indicating that Wnt/β-catenin pathway was involved in the activation of HSCs (Fig. 6B). To further explore the association between the anti-fibrosis effect of hAMSCs and Wnt/β-catenin signaling pathway in liver fibrosis, the expression levels of several proteins involved in Wnt/β-catenin signaling pathway in LX-2 were examined by western blot. As shown in Fig. 6C, both hAMSCs and hAMSC-CM downregulated the phosphorylation of GSK3β and the expression of β-catenin and α-SMA. Knockdown of the DKK-3 and DKK-1 gene with their siRNA resulted in an approximate 75% and 68% reduction in the protein levels of DKK-3 and DKK-1, respectively (Fig. 6D, E). Then, the LX-2 cells were treated with normal hAMSCs or hAMSCs transfected with DKK-3 and DKK-1 siRNA. The hAMSCs transfected with DKK-3 and DKK-1 siRNA partially lost the ability of suppressing the expressions of β-catenin and α-SMA in LX-2 cells (Fig. 6F). These results demonstrated that DKK-3 and DKK-1 derived from hAMSCs inhibited LX-2 cell activation through blocking canonical Wnt signaling pathway.

### hAMSCs-derived IGFBP3 inhibited HSCs activation through blocking PI3K/AKT-Wnt/β-catenin signaling pathway

**Fig. 7:**
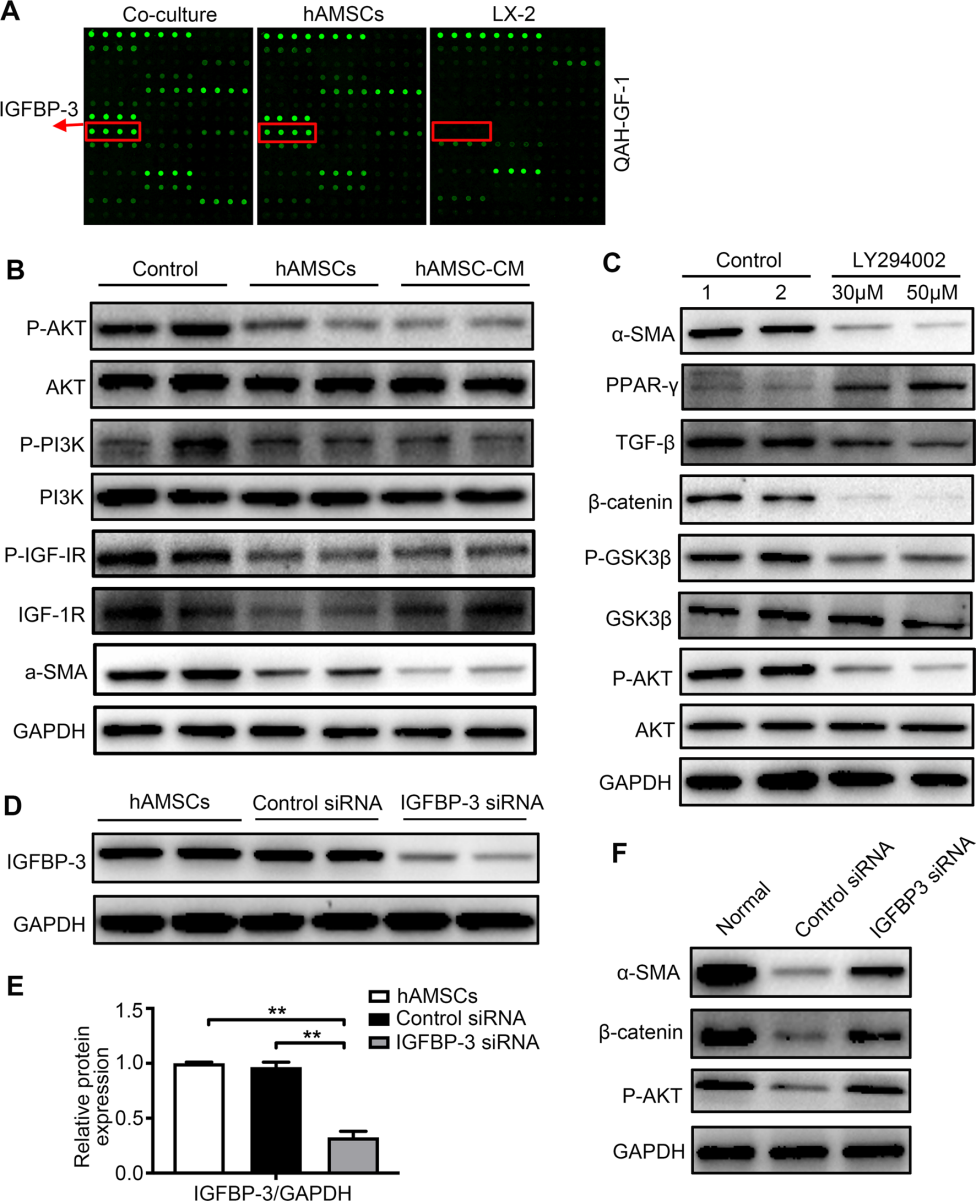
IGFBP-3 derived from hAMSCs inhibits LX-2 cell activation through blocking PI3K/AKT-Wnt/β-catenin signaling pathway. **A** Representative array images are shown (n = 4). IGFBP-3 is highlighted with red boxes and arrows. **B** LX-2 cells were treated with normal medium (control), hAMSCs, and hAMSC-CM. The expression levels of α-SMA, IGF-1R, P-IGF-1R, PI3K, P-PI3K, AKT, and P-AKT in LX-2 cells of different groups were analyzed by western blot after 48 h of treatment. **C** Western blot detection of AKT, P-AKT, GSK3β, P-GSK3β, β-catenin, TFG-β, PPAR-γ, and α-SMA protein expression in LX-2 after LY294002 treatment. The normal LX-2 was used as control. **D** LX-2 was cultured for 48 h in the presence of control siRNA or IGFBP-3 siRNA. Cell lysates were analyzed by western blot using antibodies against IGFBP-3. **E** Quantitative analysis of the expression of IGFBP-3 in hAMSCs cells of different groups as in (**D**). **F** Western blot analysis showed that the expression of P-AKT, β-catenin, and α-SMA was increased in IGFBP-3 siRNA group when compared with control siRNA group

IGFBP3 was the cytokine with the highest concentration in the co-culture group and hAMSCs group but was not detectable in the LX-2 group (Fig. 7A), which controls the bioavailability and bioactivity of IGF. IGF/IGF-1R has the ability to activate PI3K/AKT pathway by triggering receptor and intracellular targets phosphorylation [24]. As shown in Fig. 7B, both hAMSCs and hAMSC-CM downregulated the phosphorylation of IGF-1R, PI3K, and AKT. The activated LX-2 cells were treated with or without PI3K inhibitor LY294002 (30 μM and 50 μM) to confirm whether the PI3K/AKT pathway is necessary for the activation of HSCs. The results showed that LY294002 inhibited the phosphorylation of AKT and GSK3β, and the expressions of β-catenin, TGF-β, and α-SMA were decreased in LX-2 cells treated with LY294002 compared to controls (Fig. 7C). These results indicated that PI3K/AKT-mediated the activation of HSCs was dependent on inhibition of Wnt/β-catenin signaling pathway. To silence the IGFBP-3 gene, the siRNA targeting IGFBP-3 was transfected into normal hAMSCs and resulted in an approximate 68% reduction in IGFBP-3 protein levels (Fig. 7D, E). Then, LX-2 cells were treated with normal hAMSCs or hAMSCs transfected with IGFBP-3 siRNA. The results showed that hAMSCs transfected with IGFBP-3 siRNA partially lost the ability of suppressing the phosphorylation of AKT and the expressions of β-catenin and α-SMA in LX-2 cells (Fig. 7F). These results indicated that IGFBP-3 derived from hAMSCs inhibits LX-2 cell activation through blocking PI3K/AKT-Wnt/β-catenin signaling pathway.

In addition, the results also showed that hAMSCs and hAMSC-CM also downregulated the phosphorylation of ERK1/2 and JNK (Additional file 2: Figure S1A and S1C). Next, LX-2 cells were treated with or without ERK1/2 inhibitor PD98059 (30 μM and 50 μM) and JNK inhibitor SP600125 (10 μM and 25 μM). The results showed that PD98059 and SP600125 inhibited the phosphorylation of ERK1/2 and JNK, respectively (Additional file 2: Figure S1B and S1B). However, no significant difference in the expression of α-SMA was observed in the inhibitor group compared to the control group.

## Discussion

Liver fibrosis is an intense repair as well as cicatrization mechanism-related disorder. Cirrhosis is the end stage of liver fibrosis with a high global morbidity and mortality [25]. It has been demonstrated that MSCs have the potential for liver fibrosis therapy. In contrast, some studies reported that MSCs contributed to the progression of liver fibrosis [26, 27]. In recent years, hAMSCs have been considered as one of the most promising stem cells in the field of regenerative medicine clinically [19]. However, the exact effects and the underlying mechanism of hAMSCs on liver fibrosis are not explored. Extracellular matrix (ECM) produced by activated HSCs is mainly composed of type I and type III collagen [28]. In the present study, we observed that hAMSCs significantly decreased the collagen deposition in mouse liver fibrosis models induced by the injection of CCl_4_. Consistent with these results, HSCs treated with hAMSCs and hAMSC-CM remarkably reduced the expressions of collagen I and collagen III proteins.

A few studies reported that the MSCs inhibited liver fibrosis and improved liver function by differentiating into liver cells in the damaged tissue [12, 29]. In contrast, numerous studies demonstrated that MSCs exerted their anti-fibrotic effects in liver fibrosis by inhibiting the activation of HSCs through their paracrine factors [30]. To further explore the antifibrotic mechanisms of hAMSCs in liver fibrosis, the hAMSCs were labeled with MAB1281 and CD90 for monitoring the fate of the cells in mice with liver fibrosis. Our result showed that the MAB1281-positive cells also expressed CD90 that is a marker of MSC, indicating that the positive cells in mouse liver tissues represented the hAMSCs. These results indicated that hAMSCs inhibited the activation of HSCs through their paracrine mechanism since both hAMSCs or hAMSCs-CM has a similar efficiency to the inhibition of HSCs.

MSC-derived secretome has been proved to be safer and equally effective reagent in liver regeneration [31]. Huang et al. observed that CM derived from bone marrow (BM)-MSC also inhibited liver fibrosis induced by TGF-β-activated HSCs [32]. In the present study, we observed that both hAMSCs and hAMSC-CM inhibited the activation of HSCs and reduced the expression of collagen I and collagen III. A complex network of cytokines and pathways is responsible for HSC activation and induction of fibrogenic alterations [18]. Studies showed that several MSCs-secreted cytokines such as HGF [33], VEGF-A [34], MFGE8 [15], and IL-10 [14] inhibited the activation of HSCs. To further elucidate the anti-fibrotic mechanisms of the cytokine-secreted by hAMSCs, an antibody array was used to determine the potential molecules. Our results showed that 56 cytokines were highly expressed in the co-culture group and hAMSCs group compared to LX-2 group (Table 2), among which the top 11 cytokines with the highest expressions were IGFBP-3, PAI-1, TSP-1, VEGF-R1, Dkk-3, TFPI, ANGPTL-4, Thrombospondin-2, TIMP-1, Nidogen-1, and DKK-1, respectively. VEGF-R1, ANGPTL-4, Nidogen-1 have not been reported to be associated with the occurrence of liver fibrosis. PAI-1, the potent inhibitor of fibrinolytic activity, was significantly elevated in fibrotic liver [35]. TSP-1 is an endogenous activator for TGF- β activation, in which the increased TSP1 expression and TGF-β levels have been observed in livers from patients suffered from the congenital hepatic fibrosis [36]. Tetsuo et al. reported that Thrombospondin-2 might be a useful biomarker for advanced fibrosis diagnosis in patients with non-alcoholic liver disease [37]. TIMP-1 was not essential for hepatic fibrogenesis in mice [38].

In comparison, IGFBP-3, an insulin growth factor transport protein, was downregulated in patients with chronic hepatitis C [39]. Marcelo et al. observed that the serum IGFBP-3 level in patients with cirrhosis was lower than that of healthy people [40]. DKK-3 and DKK-1 are two antagonists for Wnt pathway. Jason et al. demonstrated that DKK-1 inhibited HSCs activation and liver fibrosis in animal model [41]. TFPI is a multivalent Kunitz-type serine protease inhibitor that regulates tissue factor-induced coagulation via factor Xa-dependent feedback inhibition of the tissue factor–factor VIIa complex [42]. TFPI overexpression had a protective effect on the development of fibrosis and HSC activation after partial ligation of the inferior vena cava [43].

PI3K/Akt, Wnt/β-catenin, JAK/STAT, and the Ras/Raf are the most important pathways involved in hepatic fibrosis [44]. PI3K/AKT signaling pathway is involved in numerous biological processes in a variety of cells [45], and inhibition of PI3K/AKT signaling pathway exerted an antifibrotic effect [46, 47]. IGF signaling pathway is activated by IGF-1/IGF-2 binding to IGF-1R in the plasma membrane, ultimately leading to activation of PI3K/AKT pathway [48]. IGFBP3 is a main mediator for IGF signaling pathway [49]. β-catenin is the pivotal signaling molecule that mediates the canonical Wnt signaling pathway [50], which is activated in the HSCs of liver fibrosis and contributed to the development of fibrosis by upregulating α-SMA expression [51]. DKK-1 and DKK-3 have been identified as the physiological inhibitors of canonical Wnt signaling pathway. Our results showed that the expression levels of IGFBP3, DKK-3, and DKK-1 were high, among which IGFBP3 was the most abundant factor in the co-culture group and hAMSCs group. Furthermore, we found that both hAMSCs and hAMSC-CM significantly inhibited the activation of IGF-1R/PI3K/AKT and Wnt/β-catenin signaling pathways. Finally, in the present study, the expression levels of IGFBP-3, DKK-3, and DKK-1 in hAMSCs were downregulated by transfection with siRNA targeting IGFBP-3, DKK-3, and DKK-1 mRNA, and the hAMSCs transfected with IGFBP3, DKK-3, and DKK-1 siRNA partially lost the ability of suppressing the activation of HSCs. As far as we know, this is the first time to report that IGFBP3, DKK-3, and DKK-1 derived from hAMSCs inhibit HSCs activation by blocking PI3K/Akt and canonical Wnt/β-catenin signaling pathways. These results demonstrated that hAMSCs-derived IGFBP-3, DKK-3, and DKK-1 inhibited the activation of HSCs by blocking Wnt/β-catenin signaling pathway.

## Conclusion

**Fig. 8:**
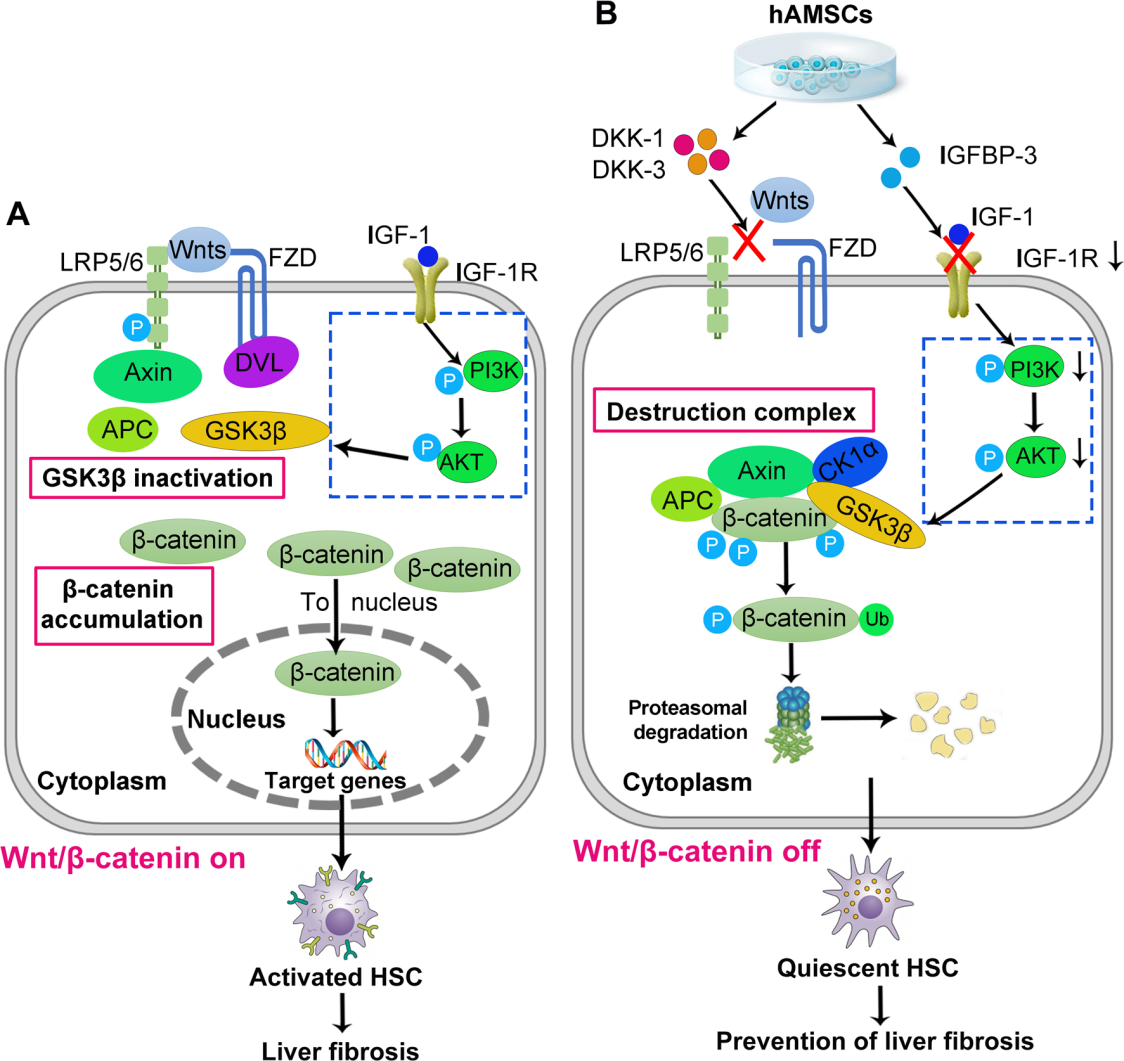
Schematic diagram of the extracellular and intracellular mechanisms of hAMSCs-derived DKK-3, DKK-1, and IGFBP3 effect on the activation of HSCs. **A** Activated HSCs: The Wnt/β-catenin signaling pathway is in the activated state (on). The Wnts act on the FZD/LRP complex on the surfaces of HSCs. Wnt-Fzd and LRP coordinate the Dvl activation, leading to the recruitment of axin to the plasma membrane. Activated Dvl then dissociates the multiprotein complex consisting of axin, APC, CK1α, and GSK3β, resulting in the inactivation of GSK3β, which can no longer phosphorylate the β-catenin. Excess free β-catenin translocates to the nucleus, causing activation of HSCs. The IGF-1R/PI3K/AKT signaling pathway is also activated in the activated state. **B** Quiescent HSCs: hAMSCs-secreted Dkk-1 and Dkk-3, two antagonists of Wnt/β-catenin signaling pathway, are capable of suppressing the binding of Wnt ligands to FZD receptors and LRP co-receptors. β-catenin is assembled and phosphorylated by the destruction complex to be further ubiquitinated (Ub) for proteasomal degradation. IGFBP-3 secreted by hAMSCs can inhibit IGF1R/PI3K/AKT signaling by sequestering IGF1, resulting in the activation of GSK3β and GSK3β signaling.

In the present study, we observed that hAMSCs significantly decreased collagen deposition, improved liver functions and inhibited HSCs activation in mice with liver fibrosis, and demonstrated that hAMSCs-derived IGFBP-3, Dkk-3, and Dkk-1 contributed to their inhibition of liver fibrosis through inhibiting HSCs activation by blocking Wnt/β-catenin signaling pathway (Fig. 8). Obviously, our findings should provide an insight in elucidating the mechanism and clinical application of hAMSCs in the treatment of liver fibrosis.

## Supplementary Information

**Additional file 1: Table S1**. The expression levels of 440 cytokines in the culture supernatant of the LX-2, hAMSCs, and co-culture group.

**Additional file 2: Fig. S1**. hAMSCs and hAMSC-CM treatment inhibited the expression of P-ERK1/2 and P-JNK in LX-2 cells. (A, C) LX-2 cells were treated with normal medium (control), hAMSCs, and hAMSC-CM. The expression levels of ERK1/2, P-ERK1/2, JNK, and P-JNK in LX-2 cells of different groups were analyzed by western blot after 48 h of treatment. (B, D) Western blot detection of P-ERK1/2, P-JNK, and α-SMA protein expression in LX-2 after PD98059 or SP600125 treatment. The normal LX-2 was used as control.

## Data Availability

The data that support the findings of this study are available from the corresponding author upon reasonable request.

## Abbreviations

HAMSCs: Human amniotic mesenchymal stem cells
CCl_4_: Carbon tetrachloride
IGFBP-3: Insulin-like growth factor binding protein-3
DKK-3: Dickkopf-3
DKK-1: Dickkopf-1
CM: Conditioned medium
HSCs: Hepatic stellate cells
ECM: Extracellular matrix
α-SMA: Alpha-smooth muscle actin
MSCs: Mesenchymal stem cells
IL-10: Interleukin-10
MFGE8: Milk fat globule-EGF factor 8
FGF4: Fibroblast growth factor 4
HGF: Hepatocyte growth factor
GFP: Green fluorescent protein
TBIL: Total bilirubin
ALT: Alanine aminotransferase
AST: Aspartate aminotransferase
ALB: Albumin
ALP: Alkaline phosphatase
siRNA: Small interfering RNA
