# Supplementary material for: Human amniotic mesenchymal stem cells-derived IGFBP-3, DKK-3, and DKK-1 attenuate liver fibrosis through inhibiting hepatic stellate cell activation by blocking Wnt/β-catenin signaling pathway in mice
Source: Stem Cell Res Ther. 2022 Jun 3;13:224. doi: 10.1186/s13287-022-02906-z (PMC9166579; doi:10.1186/s13287-022-02906-z)
Supplement: Supplementary file 1 — Additional file 1: Table S1. The expression levels of 440 cytokines in the culture supernatant of the LX-2, hAMSCs, and co-culture group. [file 13287_2022_2906_MOESM1_ESM.docx]

|  | **Concentration (pg/mL)** | | |
| --- | --- | --- | --- |
| **Cytokines** | **Mean_Coculture** | **Mean_hAMSCs** | **Mean_LX-2** |
| Periostin | 138838.3911 | 22592.24199 | 183410.6176 |
| IGFBP-3 | 56932.3678 | 45401.20049 | 0 |
| PAI-1 | 51027.64547 | 47508.89115 | 30.67226692 |
| TSP-1 | 40421.80083 | 46888.60088 | 2083.020038 |
| VEGF R1 | 33031.62889 | 44504.7282 | 38.3324137 |
| Dkk-3 | 33000.73426 | 37200.47249 | 0 |
| TFPI | 24775.95059 | 1248.839624 | 0.619501692 |
| ANGPTL4 | 23829.98389 | 28309.84487 | 4435.142538 |
| ADAMTS13 | 23474.29562 | 1514.229186 | 25466.92067 |
| Thrombospondin-2 | 20881.28501 | 19240.45644 | 1444.047002 |
| Follistatin-like 1 | 19983.17572 | 711.149994 | 19925.96459 |
| TIMP-1 | 19493.73353 | 11697.53576 | 35.7873645 |
| NCAM-1 | 19313.54347 | 16793.46412 | 16521.8675 |
| L1CAM-2 | 18273.60489 | 0 | 24693.12434 |
| Nidogen-1 | 17032.03501 | 19445.91645 | 3820.074253 |
| DKK-1 | 12279.79182 | 11930.42348 | 30.32114266 |
| TIMP-2 | 11249.62613 | 9685.848476 | 4.123643825 |
| CHI3L1 | 10582.86228 | 12115.05258 | 0 |
| CD6 | 10523.38763 | 166.7626779 | 12895.83512 |
| Syndecan-3 | 9511.832233 | 0 | 21707.61651 |
| GROa | 8670.940454 | 9143.290003 | 4477.091746 |
| bIG-H3 | 7847.162115 | 9048.114522 | 0 |
| B2M | 7678.843668 | 8007.653905 | 54.90541142 |
| Fractalkine | 7597.538165 | 314.597174 | 7985.071305 |
| IGFBP-6 | 6284.869031 | 7002.614248 | 0 |
| GITR L | 6136.946223 | 0 | 9514.593386 |
| LRP-6 | 5899.491208 | 170.7751993 | 8477.687672 |
| WISP-1 | 5837.419117 | 0 | 8856.560909 |
| Ferritin | 5650.665231 | 6498.565551 | 258.6603143 |
| Decorin | 5473.517788 | 6627.550399 | 2902.522066 |
| PF4 | 5442.824963 | 4101.662534 | 133.646572 |
| MMP-1 | 4936.144319 | 4346.030104 | 29.940655 |
| Albumin | 4876.006817 | 11391.93591 | 21049.29406 |
| sFRP-3 | 4721.265277 | 4568.186989 | 6034.337943 |
| LRIG3 | 4282.157503 | 1321.340528 | 8713.731497 |
| FLRG | 4269.599157 | 4881.149844 | 886.0902286 |
| IL-6 | 3975.143781 | 3921.847936 | 3.180008485 |
| BMPR-IA | 3717.727731 | 0 | 6980.187591 |
| APRIL | 3716.089317 | 520.301425 | 4101.452503 |
| MMP-2 | 3645.832862 | 1893.390413 | 429.4984614 |
| DNAM-1 | 3600.166397 | 282.6092633 | 3702.08951 |
| uPAR | 3409.932352 | 3931.905869 | 0 |
| CA19-9 | 3381.193656 | 2881.310063 | 1102.400163 |
| CD48 | 3250.958714 | 1401.877516 | 1896.947858 |
| Legumain | 3235.955439 | 5166.152115 | 0 |
| Pentraxin 3 | 3201.645922 | 3614.372483 | 6.934564818 |
| HGF | 3076.933415 | 2381.403175 | 0.079359219 |
| G-CSF | 2907.378183 | 23869.67619 | 9.46434095 |
| Ck beta 8-1 | 2872.978336 | 406.8603166 | 2373.561676 |
| B7-H1 | 2803.360395 | 51.24205814 | 2817.781507 |
| TGFb1 | 2736.077238 | 883.5375293 | 2486.779748 |
| DPPIV | 2539.749493 | 551.4466103 | 0 |
| ENA-78 | 2450.070326 | 4644.609358 | 46.0612987 |
| IGFBP-4 | 2357.947883 | 1964.505085 | 0 |
| LAP(TGFb1) | 2263.679658 | 2133.156608 | 48.40492963 |
| Prolactin | 2150.374679 | 3325.154427 | 1629.953336 |
| Thyroglobulin | 2125.273787 | 1430.570946 | 1285.75885 |
| RBP4 | 2062.022266 | 1864.392887 | 34.75823158 |
| Angiotensinogen | 1849.387204 | 23.11903042 | 7413.77651 |
| gp130 | 1782.554108 | 1057.827792 | 26.06557417 |
| Galectin-1 | 1749.726114 | 831.2251198 | 5716.313429 |
| MEPE | 1727.538346 | 0 | 3042.803028 |
| AMICA | 1646.101071 | 7198.751756 | 2339.801177 |
| Follistatin | 1596.634068 | 1297.13307 | 2934.200267 |
| PDGF-AA | 1570.572118 | 199.795467 | 1505.698316 |
| CTLA4 | 1552.47379 | 1143.796665 | 21.52788958 |
| IL-11 | 1469.715536 | 1297.520964 | 37.76951402 |
| Cadherin-11 | 1440.781682 | 0 | 1926.929178 |
| GRO | 1395.887705 | 2214.43841 | 0 |
| BMPR-II | 1381.749416 | 61.59545659 | 1939.631735 |
| Galectin-3 | 1380.709054 | 1383.173842 | 0 |
| Persephin | 1327.945579 | 0 | 4503.358682 |
| IL-21 | 1209.640755 | 542.5699298 | 353.4675817 |
| DR3 | 1173.009088 | 545.6503935 | 808.1661479 |
| MMP-3 | 1107.167868 | 978.3551104 | 41.95096733 |
| Mer | 1088.396321 | 453.2838588 | 0 |
| GASP-1 | 1042.613813 | 947.9244103 | 995.4012699 |
| MMP-10 | 1027.558414 | 930.4165444 | 0.748626507 |
| NSE | 1019.089452 | 1201.459356 | 53.20761037 |
| DcR3 | 954.7013785 | 1040.192828 | 0 |
| GCP-2 | 949.5248561 | 1984.953832 | 57.43712661 |
| CNTF | 939.8012079 | 0 | 2230.103047 |
| aFGF | 895.1357731 | 165.3947424 | 677.997499 |
| bFGF | 873.9399526 | 423.0562029 | 853.904113 |
| IGFBP-5 | 871.6939467 | 0 | 0 |
| IL-1 F8 | 862.9746155 | 399.3486518 | 2.446001288 |
| FAP | 808.5513654 | 1533.354319 | 0 |
| ACE-2 | 795.1578678 | 598.8120039 | 592.0730189 |
| FGF-7 | 778.3293691 | 865.6898357 | 0 |
| Cystatin B | 770.1819063 | 1310.286935 | 3.722226528 |
| uPA | 746.3596929 | 1128.005703 | 0.352235285 |
| Insulin | 696.0480866 | 177.5141843 | 1049.152219 |
| BMP-5 | 673.3098762 | 308.1980241 | 285.9621545 |
| BCAM | 667.6812718 | 851.7541275 | 95.80577077 |
| RGM-B | 664.4555749 | 0 | 1232.808492 |
| Angiostatin | 644.8825935 | 659.7580646 | 862.4856424 |
| Fetuin A | 618.0448213 | 316.8160843 | 162.2783759 |
| Lymphotactin | 596.1566453 | 532.8344786 | 331.798771 |
| MCP-1 | 595.2255118 | 595.3578171 | 2.784970381 |
| ADAM9 | 588.5333371 | 918.4646244 | 104.9219854 |
| IL-10 Ra | 576.2063913 | 116.2620456 | 0 |
| Thrombomodulin | 560.3082502 | 144.2274444 | 0 |
| Activin A | 560.1542887 | 1357.888614 | 138.9795245 |
| ANG-1 | 556.5796786 | 1709.999669 | 3.262534935 |
| Midkine | 540.038388 | 774.612734 | 11.18265853 |
| MCP-3 | 536.2496514 | 577.5162488 | 0 |
| Cystatin C | 489.9678272 | 855.6066023 | 12.78700818 |
| SDF-1a | 486.459271 | 4.938482003 | 431.6929771 |
| SCF R | 483.8017963 | 402.8085761 | 0 |
| ANG-2 | 448.517265 | 2515.46714 | 459.3252059 |
| MIF | 448.179106 | 664.548602 | 338.3028499 |
| CEACAM-5 | 428.6456514 | 374.7011869 | 122.9410753 |
| IL-1 F9 | 426.8876149 | 303.6789428 | 141.1688168 |
| Furin | 418.8434428 | 162.4232554 | 234.6969323 |
| EMMPRIN | 412.651994 | 347.3121665 | 76.69804627 |
| IFNab R2 | 411.3828033 | 2.312685173 | 385.4529816 |
| Marapsin | 397.2022369 | 357.5719418 | 106.3995523 |
| TPO | 387.9700565 | 133.6653168 | 813.8220712 |
| ICAM-1 | 384.7978733 | 410.3974754 | 77.45116459 |
| CXCL16 | 384.2995872 | 611.1009215 | 23.84031576 |
| Nectin-4 | 363.7398231 | 0 | 1280.561944 |
| S100A8 | 349.0652384 | 12.67460645 | 690.6607901 |
| Procalcitonin | 334.0477159 | 266.465864 | 25.30405452 |
| TNF RI | 330.4061239 | 191.8122683 | 5.801326368 |
| Angiogenin | 323.0870776 | 364.3062457 | 0.434595079 |
| IL-8 | 297.1578354 | 257.0999096 | 0.71535184 |
| IL-1 R3 | 287.8608894 | 106.5381557 | 673.6371267 |
| TACE | 263.7917692 | 382.3309309 | 164.7374377 |
| ICOS | 254.7847653 | 0 | 56.90974017 |
| VEGF | 248.9092902 | 0.239222767 | 8.117619029 |
| IL-17E | 244.3423567 | 0 | 539.1602677 |
| Galectin-2 | 236.7830089 | 716.1040295 | 182.9804126 |
| IL-13 R1 | 233.4413892 | 160.9319189 | 362.7551632 |
| Tie-1 | 227.0701273 | 19.49013582 | 809.3432558 |
| IL-23 | 223.6129937 | 205.0444723 | 198.9342873 |
| VEGF-C | 217.9682498 | 121.5056078 | 45.60550059 |
| FOLR1 | 217.408611 | 72.12334583 | 0 |
| ULBP-2 | 216.3856504 | 0 | 1172.936189 |
| Layilin | 215.6953522 | 265.9848876 | 0 |
| IGF-1 | 198.1095348 | 191.8453025 | 73.44018225 |
| B7-H3 | 186.0186984 | 141.0565111 | 26.89637238 |
| hCGb | 172.5451544 | 182.180087 | 63.58784871 |
| Syndecan-1 | 170.3334194 | 382.9497652 | 225.6656168 |
| IL-1 R6 | 170.0531807 | 0 | 102.7845057 |
| IL-1 F7 | 167.4921644 | 47.50149014 | 0 |
| Aggrecan | 165.7332727 | 0 | 589.6405438 |
| DLL1 | 161.6694965 | 141.3743155 | 183.895171 |
| Cadherin-4 | 151.5920064 | 958.9829045 | 137.0171783 |
| BMP-7 | 148.9507368 | 204.0636252 | 0 |
| IL-27 | 148.3012803 | 271.7844318 | 95.71161206 |
| IL-13 R2 | 142.9384155 | 113.7757811 | 58.49363335 |
| TNF RII | 142.4468137 | 87.23426677 | 2.353987597 |
| GDF-15 | 137.6543253 | 157.319001 | 0.120334872 |
| TF | 133.4163397 | 129.4223131 | 0 |
| TLR4 | 123.6437374 | 628.2104048 | 61.36827361 |
| CRTAM | 123.2470325 | 127.2985969 | 52.50101735 |
| ROBO3 | 115.4740209 | 5.603773018 | 327.9934573 |
| Transferrin | 114.7562697 | 32.55766969 | 0 |
| MIP-1a | 112.0357676 | 71.92051417 | 4.137382553 |
| Desmoglein 2 | 111.6293369 | 35.11666594 | 39.56885254 |
| CA125 | 110.6128993 | 729.7187859 | 276.7344748 |
| DR6 | 110.1896341 | 152.1378741 | 2.372000832 |
| CD27 | 107.2378605 | 3.1963705 | 184.7484229 |
| ADAM8 | 100.0174758 | 35.13055097 | 67.668574 |
| IL-15 | 99.58749743 | 120.7469282 | 40.31599941 |
| TSH | 97.34937858 | 115.2277733 | 8.525150347 |
| IL-17F | 97.01341285 | 654.1651357 | 144.7211683 |
| ANGPTL3 | 95.50380188 | 89.12620866 | 23.31401804 |
| Clusterin | 92.08109662 | 59.06317118 | 16.16019413 |
| CD14 | 90.50325496 | 58.50921773 | 0 |
| EGF R | 89.70262936 | 88.0693361 | 5.321518378 |
| GH | 88.82282998 | 49.80566232 | 62.30890081 |
| E-Cadherin | 85.30846307 | 48.45456784 | 223.5237828 |
| IL-13 | 81.63056046 | 81.2948408 | 43.89429938 |
| Galectin-9 | 79.17386241 | 269.8873855 | 60.19487439 |
| CD99 | 77.09975806 | 129.4976933 | 3.986451109 |
| Trappin-2 | 76.26783006 | 82.27742985 | 0 |
| Cadherin-13 | 76.17879229 | 58.08838866 | 20.18107231 |
| Pref-1 | 74.19050313 | 0 | 1.266186689 |
| Cathepsin L | 72.69901069 | 136.6542139 | 15.53038791 |
| SDF-1b | 72.31182129 | 30.91853793 | 64.85505994 |
| NrCAM | 72.26870263 | 52.62583482 | 52.60538811 |
| CTACK | 68.83402848 | 43.99664664 | 29.86757811 |
| OPN | 66.80792024 | 3568.783042 | 37.36887408 |
| Adipsin | 66.75769257 | 96.40747616 | 51.64585143 |
| TACI | 64.32926692 | 0 | 13.39179389 |
| IL-32 alpha | 63.96536811 | 0 | 152.3711114 |
| CD84 | 57.72164928 | 0 | 65.38795389 |
| HCC-4 | 57.09710206 | 55.3354575 | 16.61058714 |
| CF XIV | 55.05736926 | 117.7620348 | 8.124510117 |
| IGFBP-2 | 54.41051974 | 56.78925864 | 0 |
| NOV | 52.79749262 | 74.65972345 | 0.603929434 |
| MIP-1b | 52.16491034 | 17.68625854 | 0.421178652 |
| Cathepsin B | 50.89831031 | 2959.609056 | 0 |
| IL-20 | 49.24667801 | 218.1903213 | 36.0058958 |
| IGF-1R | 49.18740882 | 104.6875866 | 90.06506564 |
| IL-2 Rb | 48.67538119 | 25.43762746 | 63.89442478 |
| EDA-A2 | 48.42053682 | 50.27200869 | 5.12694654 |
| TRANCE | 46.20854331 | 0 | 6.035045683 |
| CD229 | 45.60864009 | 12.52533803 | 2.917049138 |
| BMP-4 | 44.66669809 | 37.44047928 | 67.14569306 |
| TIMP-4 | 43.88136619 | 88.32248485 | 3.631868783 |
| BMPR-IB | 41.76738 | 16.92872142 | 44.81895883 |
| TREM-1 | 41.7206027 | 55.74710915 | 44.20429185 |
| MMP-9 | 41.08396692 | 80.20544913 | 16.62250371 |
| Adiponectin | 39.97753873 | 109.1127115 | 254.1573258 |
| ICAM-2 | 39.8434219 | 138.3057166 | 127.3278076 |
| GASP-2 | 37.38650812 | 0 | 7.297337483 |
| SOST | 37.33381983 | 31.66308679 | 51.54608534 |
| FGF-21 | 37.20127897 | 143.2910064 | 29.79400231 |
| OPG | 36.86088137 | 29.99479415 | 10.07052715 |
| Granulysin | 36.57914525 | 56.30983122 | 98.95719931 |
| Siglec-10 | 36.04777617 | 0 | 0 |
| XEDAR | 34.23820483 | 0 | 48.13144019 |
| Eotaxin-3 | 32.72653473 | 27.6526518 | 19.91338453 |
| LIF | 32.47907967 | 78.1369999 | 38.41844395 |
| IL-1 RII | 32.02209373 | 51.24619461 | 30.85932927 |
| 6Ckine | 31.81724715 | 0 | 5.09401005 |
| IGFBP-1 | 31.79211879 | 29.09795092 | 4.22281481 |
| IL-34 | 31.64828507 | 13.83949087 | 0 |
| ESAM | 29.81626653 | 43.34162534 | 60.002573 |
| IL-2 | 28.31068344 | 29.56568528 | 13.69407502 |
| 2B4 | 28.1902965 | 18.80801367 | 6.989079491 |
| ADAM12 | 27.82855684 | 21.70985365 | 170.6711168 |
| Fcg RIIBC | 27.52897213 | 55.77550811 | 116.079499 |
| b-NGF | 27.25402853 | 2.395613537 | 45.56947265 |
| IL-2 Ra | 27.13789556 | 25.48011736 | 42.34789098 |
| TGFb2 | 26.96324052 | 4.959974243 | 59.14344963 |
| FGF-4 | 26.63795566 | 0 | 0 |
| NRG1-b1 | 25.71172944 | 0 | 0 |
| OSM | 25.14340077 | 14.87216986 | 5.498498973 |
| PDGF-AB | 24.01209043 | 8.356720942 | 21.64368808 |
| HGF R | 23.39125622 | 33.97900995 | 0 |
| IL-3 | 22.74676418 | 44.21773503 | 23.82791958 |
| CD97 | 22.3186603 | 0 | 0.031541307 |
| BDNF | 22.16155568 | 13.99734464 | 3.425868207 |
| Leptin | 21.33454882 | 25.84102461 | 8.649244462 |
| IL-12p40 | 20.96775558 | 32.68494099 | 0 |
| CRP | 20.86726569 | 24.62793075 | 33.09002915 |
| ErbB4 | 20.86402517 | 8.975232671 | 6.410012646 |
| Troponin I | 20.80344926 | 19.49790844 | 15.13454503 |
| CD163 | 20.67105111 | 0 | 27.37543533 |
| HAI-2 | 20.15045937 | 2.799058341 | 4.576694112 |
| TNFb | 19.26105263 | 18.54213647 | 11.16572664 |
| IL-5 Ra | 18.65345767 | 0 | 0 |
| IL-15 R | 18.46016244 | 0 | 27.76853766 |
| ANG-4 | 18.28071211 | 29.0602674 | 4.05525966 |
| RANTES | 17.78717734 | 15.15047114 | 23.62171855 |
| Cathepsin S | 16.335078 | 37.16088749 | 3.262534935 |
| MMP-8 | 15.88229106 | 38.13436984 | 1.486285215 |
| IL-18 BPa | 15.3845233 | 16.19102758 | 18.64227147 |
| BAFF | 15.15057053 | 0 | 1.429222984 |
| AR | 15.0076108 | 18.57345513 | 2.036740287 |
| Siglec-7 | 14.97644234 | 0 | 48.73447491 |
| CXCL14 | 14.95217363 | 0 | 1.662002637 |
| PSA-free | 14.86442777 | 18.29406653 | 7.65664002 |
| Tie-2 | 14.4026536 | 32.18150592 | 40.00463194 |
| IFNg | 14.36549172 | 15.27579677 | 6.552173163 |
| LDL R | 14.04723536 | 18.12062692 | 0 |
| IL-31 | 13.46282754 | 217.845583 | 45.02148191 |
| GDNF | 12.66311429 | 2.075453581 | 0.07307102 |
| MIP-3a | 12.49074924 | 1.137551821 | 0.931799233 |
| MMP-13 | 11.8820423 | 17.70726152 | 1.056148994 |
| IL-7 | 11.17333285 | 12.65830942 | 4.269613788 |
| MDC | 10.58613194 | 13.21345357 | 7.087509723 |
| IL-1a | 10.54623638 | 20.96385641 | 11.94967654 |
| Syndecan-4 | 10.38837432 | 25.85824841 | 0 |
| LIMPII | 10.31189673 | 71.56306122 | 0 |
| Thrombospondin-5 | 9.946404109 | 0 | 62.18252276 |
| MCP-2 | 9.790977961 | 4.605603064 | 0.856778963 |
| BMP-2 | 9.740889992 | 0 | 3.73290123 |
| Renin | 9.625343258 | 0 | 35.90040791 |
| Shh-N | 8.725247714 | 6.017186158 | 12.47198573 |
| Neprilysin | 8.588451264 | 38.30879959 | 17.10265505 |
| TNFa | 8.517636531 | 10.12366432 | 6.090885513 |
| IGF-2 | 8.253921967 | 602.0606861 | 13.87912197 |
| Cystatin E M | 8.082441262 | 6.034093457 | 0.795394081 |
| MCP-4 | 7.661501276 | 8.094295768 | 4.665885698 |
| IP-10 | 7.637124012 | 6.05022591 | 2.774191807 |
| JAM-A | 7.497689284 | 10.53218011 | 1.769172222 |
| EG-VEGF | 6.927375056 | 3.599838288 | 1.031365047 |
| Siglec-5 | 6.641968217 | 6.579408778 | 4.736293532 |
| BCMA | 6.525294483 | 33.58730978 | 18.00273 |
| Cystatin A | 6.331248049 | 0 | 0 |
| FGF-9 | 5.681726169 | 0.254791413 | 1.465456459 |
| TGFb3 | 5.413616536 | 0 | 0 |
| I-TAC | 5.256923922 | 2.983184282 | 5.053356685 |
| IL-1b | 5.226890105 | 2.465260909 | 0.54862113 |
| TLR2 | 5.123774883 | 2.300039365 | 4.603313633 |
| IL-5 | 4.86738381 | 6.130736659 | 2.11916595 |
| WIF-1 | 4.717834924 | 1.728923427 | 0 |
| IL-4 | 4.655172414 | 4.857765734 | 1.70084053 |
| ST2 | 4.636142509 | 1.010954867 | 6.128053265 |
| 4-1BB | 4.325610747 | 81.25563155 | 21.62146519 |
| GM-CSF | 4.12270316 | 6.038762906 | 0.666301845 |
| G-CSF R | 4.046240724 | 0 | 0.40800843 |
| Siglec-9 | 4.041088335 | 0.650154202 | 2.668311665 |
| LOX-1 | 3.480656599 | 6.299387637 | 0 |
| HB-EGF | 3.402437078 | 3.567557613 | 1.120155454 |
| ULBP-1 | 3.351670148 | 19.32325465 | 3.82099422 |
| NAP-2 | 3.324602088 | 8.887580263 | 5.874398954 |
| IL-33 | 3.287173056 | 7.89174423 | 0 |
| Osteoactivin | 3.277180269 | 8.233730909 | 2.336636471 |
| TIM-1 | 3.110937705 | 14.97380873 | 0.621912118 |
| MCSF R | 3.043976613 | 3.597595875 | 0 |
| IL-1ra | 3.042698522 | 3.420244222 | 1.126698477 |
| AgRP | 3.034788721 | 2.64199076 | 2.853021722 |
| EpCAM | 2.970064984 | 1.527945739 | 4.150346225 |
| Axl | 2.850928056 | 0.439241302 | 0 |
| AFP | 2.824871129 | 3.058225213 | 1.077548857 |
| Eotaxin | 2.820086023 | 1.754126634 | 2.883222578 |
| IL-16 | 2.808758792 | 2.719892817 | 3.767507537 |
| MIP-3b | 2.659736926 | 39.8748702 | 11.72378025 |
| Cripto-1 | 2.609976863 | 6.418336731 | 5.237131722 |
| FSH | 2.439916549 | 1.363229847 | 4.029695102 |
| MCSF | 2.4033454 | 0.592978132 | 2.047752644 |
| ALCAM | 2.27909634 | 2.02161477 | 0 |
| IL-10 | 2.250879144 | 1.76703297 | 0.945094387 |
| Testican 2 | 2.15006593 | 99.84261777 | 12.67750369 |
| TRAIL R2 | 1.875263483 | 0.822724418 | 0.806474224 |
| Galectin-7 | 1.779212346 | 119.516204 | 11.57116263 |
| I-309 | 1.48073697 | 0.40211868 | 1.28221039 |
| TSLP | 1.423851056 | 62.52010916 | 15.89994793 |
| TRAIL R4 | 1.405756327 | 2.901538206 | 4.419434902 |
| MIG | 1.214381263 | 7.271569295 | 6.679739201 |
| Eotaxin-2 | 1.177225081 | 0.472594389 | 3.454438836 |
| CD40 | 1.142763087 | 1.817011485 | 2.954857807 |
| PGRP-S | 0.779049398 | 0.356541901 | 0.152769909 |
| LIGHT | 0.631145183 | 7.565866792 | 6.83767583 |
| Kallikrein 5 | 0.629423846 | 0 | 0 |
| ErbB2 | 0.57314528 | 0.187283542 | 1.281717498 |
| Gas 1 | 0.447089184 | 0.320447325 | 0 |
| IL-6R | 0.40371915 | 0 | 1.255086907 |
| BLC | 0.382547301 | 0.198594003 | 0.362340465 |
| Kallikrein 14 | 0.362476513 | 2.299269705 | 1.492816875 |
| VEGF-D | 0.301241247 | 0.309016903 | 0.149777297 |
| IL-17 | 0.251497574 | 0.157997705 | 0.205426521 |
| Pepsinogen I | 0.250061209 | 0 | 5.14103448 |
| PIGF | 0.172526145 | 0.742855016 | 0.178560322 |
| FAS L | 0.162547737 | 1.229568391 | 8.499911072 |
| MBL | 0.137930626 | 0.355804994 | 0.305736152 |
| PDGF-BB | 0.117718951 | 0 | 0.299299003 |
| IL-12p70 | 0.09609416 | 0.004132279 | 0.116272085 |
| TGFa | 0.08870223 | 0.789107782 | 0.042018179 |
| EGF | 0.057712563 | 0.079448547 | 0 |
| MIP-1d | 0.018052163 | 1.025085522 | 0.832704453 |
| TRAIL | 0.011919815 | 0.235431083 | 0 |
| IL-17B | 0.006187812 | 3.040399344 | 71.4501375 |
| TRAIL R3 | 0.002863118 | 0 | 0 |
| DAN | 0 | 0 | 0 |
| Resistin | 0 | 6.94637322 | 46.63536905 |
| CA15-3 | 0 | 0.463466382 | 0.500178836 |
| CEA | 0 | 55.8946855 | 31.87658543 |
| IL-18 Rb | 0 | 3.959107608 | 0.059712203 |
| BMP-9 | 0 | 0 | 0 |
| C5a | 0 | 0 | 0 |
| CD200 | 0 | 0 | 0 |
| Chemerin | 0 | 0 | 0 |
| FABP2 | 0 | 0 | 0 |
| FGF-19 | 0 | 0 | 0 |
| IGF-2R | 0 | 0 | 0 |
| IL-24 | 0 | 0 | 0 |
| Notch-1 | 0 | 0 | 0 |
| PD-1 | 0 | 6.327303463 | 0 |
| Serpin A4 | 0 | 0 | 0 |
| TRAIL R1 | 0 | 0 | 0 |
| IL-17B R | 0 | 0 | 0 |
| LAG-3 | 0 | 0 | 0 |
| RANK | 0 | 0 | 0 |
| VE-Cadherin | 0 | 0 | 0 |
| CA9 | 0 | 0 | 0 |
| CD23 | 0 | 0 | 0 |
| Dkk-4 | 0 | 0 | 0 |
| Epo R | 0 | 0 | 0 |
| FGF-6 | 0 | 0 | 0 |
| IL-1 F5 | 0 | 186.3234452 | 0 |
| IL-1 F6 | 0 | 0 | 0 |
| IL-1 F10 | 0 | 0 | 0 |
| IL-1 R5 | 0 | 0 | 0 |
| IL-17C | 0 | 0 | 0 |
| IL-18 | 0 | 0 | 0 |
| Leptin R | 0 | 0 | 0 |
| MMP-7 | 0 | 0 | 0 |
| P-Cadherin | 0 | 22.76832172 | 9.370798297 |
| Prostasin | 0 | 3.867014983 | 0 |
| PSMA | 0 | 0 | 0 |
| SIGIRR | 0 | 0 | 0 |
| TGFb RIII | 0 | 0 | 0 |
| TWEAK | 0 | 0 | 0 |
| CD58 | 0 | 6.370338173 | 0 |
| CD155 | 0 | 0 | 0 |
| JAM-B | 0 | 0 | 0 |
| SLAM | 0 | 0 | 0 |
| SP-D | 0 | 0 | 0 |
| TIM-3 | 0 | 0 | 0 |
| BTC | 0 | 0 | 0 |
| CCL28 | 0 | 0 | 0 |
| HCC-1 | 0 | 0 | 1.310414195 |
| IL-9 | 0 | 0 | 0 |
| IL-28A | 0 | 0 | 0.036687836 |
| IL-29 | 0 | 0 | 0 |
| MPIF-1 | 0 | 0 | 0 |
| MSP | 0 | 0 | 0 |
| PARC | 0 | 0 | 0 |
| TARC | 0 | 0.924595849 | 0 |
| TECK | 0 | 0 | 0 |
| NGF R | 0 | 0 | 0 |
| NT-3 | 0 | 0 | 0 |
| NT-4 | 0 | 0.500046484 | 9.53707801 |
| SCF | 0 | 0 | 0 |
| VEGF R2 | 0 | 0 | 0 |
| VEGF R3 | 0 | 0 | 0 |
| B7-1 | 0 | 0 | 0 |
| CD30 | 0 | 0 | 0 |
| CD40L | 0 | 0 | 0 |
| CEACAM-1 | 0 | 0 | 0 |
| Dtk | 0 | 0 | 0 |
| Endoglin | 0 | 0.397991025 | 0 |
| ErbB3 | 0 | 0 | 0 |
| E-Selectin | 0 | 0 | 0 |
| Fas | 0 | 0 | 0 |
| Flt-3L | 0 | 0.041188556 | 0 |
| GITR | 0 | 0 | 0 |
| HVEM | 0 | 0 | 0 |
| ICAM-3 | 0 | 0 | 0 |
| Contactin-2 | 0 | 0 | 0 |
| IL-1 RI | 0 | 0 | 0 |
| IL-2 Rg | 0 | 0 | 0 |
| IL-10 Rb | 0 | 0 | 0 |
| IL-17R | 0 | 0 | 0 |
| IL-21R | 0 | 0 | 0 |
| Lipocalin-2 | 0 | 0 | 0 |
| L-Selectin | 0 | 0 | 0 |
| LYVE-1 | 0 | 0 | 0 |
| MICA | 0 | 0 | 0 |
| MICB | 0 | 0 | 0 |
| PDGF Rb | 0 | 0 | 0 |
| PECAM-1 | 0 | 0 | 0 |
| RAGE | 0 | 0.028739467 | 0 |
| VCAM-1 | 0 | 0 | 0 |
