# Supplementary figures and images for: Human amniotic mesenchymal stem cells-derived IGFBP-3, DKK-3, and DKK-1 attenuate liver fibrosis through inhibiting hepatic stellate cell activation by blocking Wnt/β-catenin signaling pathway in mice
Source: Stem Cell Res Ther. 2022 Jun 3;13:224. doi: 10.1186/s13287-022-02906-z (PMC9166579; doi:10.1186/s13287-022-02906-z)

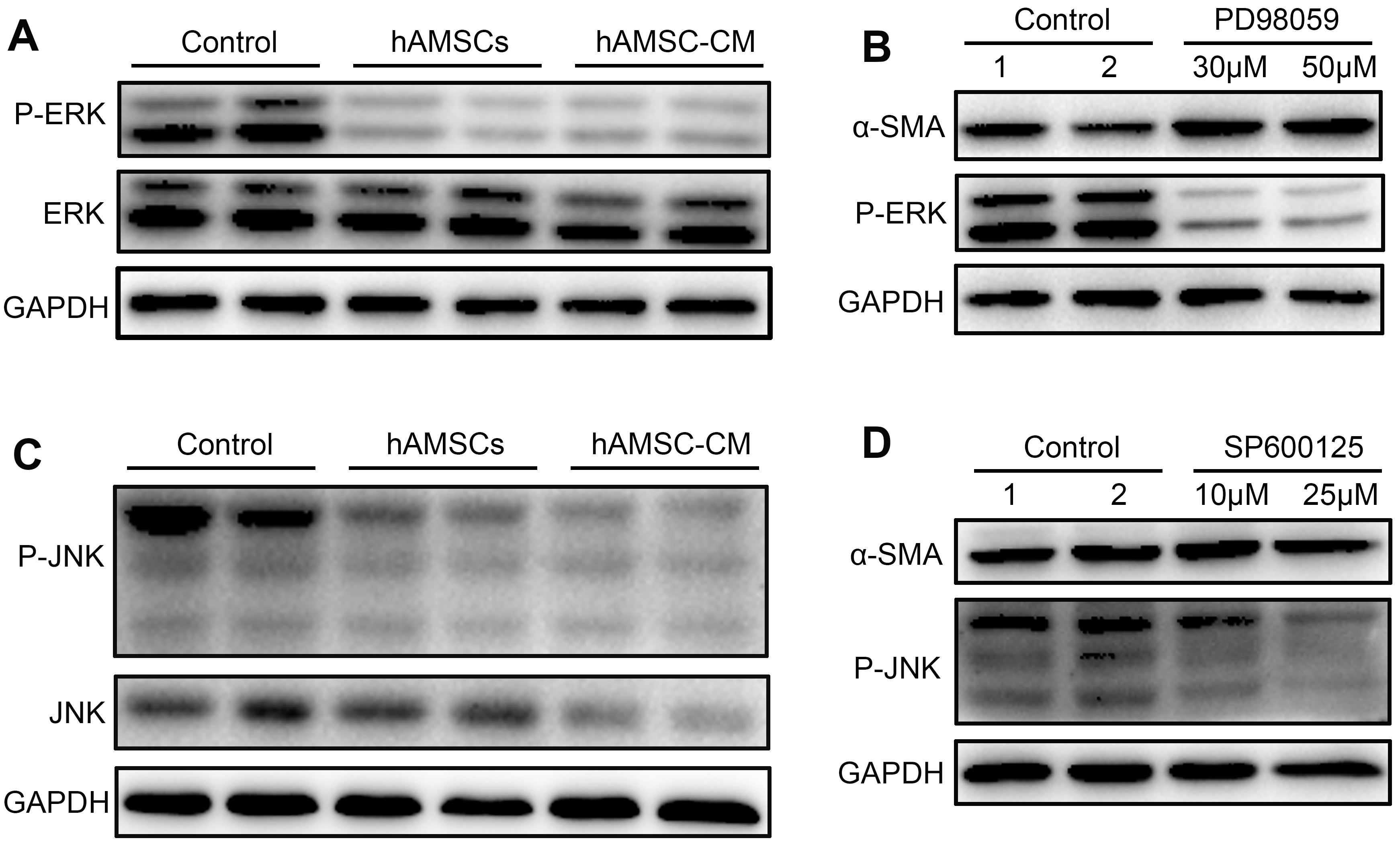

Supplement: Supplementary file 2 — Additional file 2: Fig. S1. hAMSCs and hAMSC-CM treatment inhibited the expression of P-ERK1/2 and P-JNK in LX-2 cells. (A, C) LX-2 cells were treated with normal medium (control), hAMSCs, and hAMSC-CM. The expression levels of ERK1/2, P-ERK1/2, JNK, and P-JNK in LX-2 cells of different groups were analyzed by western blot after 48 h of treatment. (B, D) Western blot detection of P-ERK1/2, P-JNK, and α-SMA protein expression in LX-2 after PD98059 or SP600125 treatment. The normal LX-2 was used as control. [file 13287_2022_2906_MOESM2_ESM.tif]
